# Conventional and Rock-Based Potassium Fertilization Improves Fennel (*Foeniculum vulgare* Mill.) Performance Under Extended Irrigation Intervals

**DOI:** 10.3390/plants15040573

**Published:** 2026-02-11

**Authors:** Ghada F. H. El-Sheref, Nevien Elhawat, A. G. M. Kenawy, Gihan A. Mohamed, Mahmoud M. A. Shabana, Tarek Alshaal

**Affiliations:** 1 Soils, Water and Environment Research Institute (SWERI), Agricultural Research Center, Giza 12619, Egyptshabanamma@gmail.com (M.M.A.S.); 2 Institute of Applied Plant Biology, Faculty of Agricultural and Food Sciences and Environmental Management, University of Debrecen, 4032 Debrecen, Hungary; 3 Department of Food Biotechnology, Albert Kázmér Mosonmagyaróvári Kar—AKMK, Széchenyi István University, Egyetem Sgr. 1, 9026 Győr, Hungary; 4 Faculty of Agriculture (for Girls), Al-Azhar University, Nasr City, Cairo 11884, Egypt; 5 Medicinal and Aromatic Plants Research Department, Horticulture Research Institute, Agricultural Research Center (ARC), Giza 12619, Egypt; 6 Soil and Water Science Department, Faculty of Agriculture, Kafrelsheikh University, Kafr El-Sheikh 33516, Egypt

**Keywords:** deficit irrigation, *Foeniculum vulgare*, rock-based fertilizers, water-use efficiency, nutrient uptake, semi-arid agriculture

## Abstract

Water scarcity and rising fertilizer costs challenge the sustainable cultivation of medicinal and aromatic plants in arid regions. This study evaluated the interactive effects of irrigation intervals (21, 28, and 35 days) and potassium sources (potassium sulfate and feldspar) and rates on growth, yield, essential oil productivity, and nutrient status of fennel (*Foeniculum vulgare* Mill.) over two consecutive seasons in Middle Egypt. Extending irrigation intervals significantly increased soil electrical conductivity while reducing soil-available potassium, whereas soil pH, organic matter, and bulk density remained unaffected. Frequent irrigation (21 days) markedly enhanced vegetative growth, yield components, seed yield, and essential oil yield, producing up to 69.7 L ha^−1^ oil compared with 50.5–52.0 L ha^−1^ under 35-day intervals. Potassium fertilization significantly improved plant performance across all irrigation regimes, with potassium sulfate at 120 kg K_2_O ha^−1^ consistently producing the highest plant height (≈173 cm), number of umbels (≈45 plant^−1^), 1000-seed weight (≈13 g), seed yield, and oil yield. Potassium sulfate at 120 kg K_2_O ha^−1^ consistently outperformed feldspar, though high-rate feldspar (572 kg K_2_O ha^−1^) significantly improved performance over the control, indicating potential as a supplementary source. Extending irrigation to 28 days reduced water application by approximately 23% compared to 21-day intervals, with acceptable yield levels when combined with adequate potassium supply. Potassium application enhanced seed and herb N, P, and K concentrations and mitigated the adverse effects of prolonged irrigation intervals, particularly under moderate water stress (28 days). Significant irrigation × potassium interactions confirm that optimal potassium nutrition improved water-use efficiency and reproductive performance. Overall, integrating frequent or moderately extended irrigation with an adequate potassium supply—especially soluble potassium sulfate—offers an effective strategy to sustain fennel productivity and essential oil yield under water-limited conditions.

## 1. Introduction

Fennel (*Foeniculum vulgare* Mill.) is a widely cultivated medicinal and aromatic plant in Egypt, appreciated for its dual role in food flavoring and phytopharmaceutical applications. The plant belongs to the family Apiaceae, and its fruits (commonly referred to as seeds) yield essential oil rich in phenylpropanoids (e.g., anethole, fenchone, estragole), monoterpenes, and other bioactive compounds [1]. Recent studies continue to confirm its significant pharmacological potential, including antioxidant, antimicrobial, anti-inflammatory, gastrointestinal, and hepatoprotective activities [2,3].

In Egypt, fennel is frequently grown in Middle Egypt, particularly in governorates such as Beni-Suef, Minia, and Assiut. Its fruit and essential oil are integral to various industries—from bakery and confectionery to pharmaceuticals and traditional medicine—making it a high-value crop for both farmers and processors [4]. However, agriculture in Egypt faces mounting challenges due to water scarcity, driven by diminishing Nile inflows, climate change, and population growth. The agricultural sector continues to consume the majority of available freshwater, creating urgent pressure to optimize water use and ensure sustainability. Under such constraints, improving water-use efficiency (WUE) is critical [5]. One of the strategies receiving increasing attention is deficit irrigation or extended irrigation intervals, which can substantially reduce water consumption if carefully managed [6].

From a crop physiological perspective, moderate water stress can sometimes be exploited to enhance productivity per unit of water rather than per unit of land. Deficit irrigation has been shown to improve WUE by reducing non-productive water losses while maintaining acceptable yields, particularly in aromatic and medicinal plants where biomass is not the sole economic target [7]. In fennel and related Apiaceae species, controlled water limitation has been reported to alter assimilate partitioning and stimulate the accumulation of secondary metabolites, often resulting in higher essential oil concentration and modified oil composition, even when fruit yield is slightly reduced [8]. Such responses are especially relevant under arid and semi-arid conditions, where irrigation scheduling based on longer intervals can improve irrigation efficiency and economic returns if stress thresholds are not exceeded [9]. Therefore, understanding fennel’s yield–quality–water relationships under extended irrigation intervals is essential for designing irrigation strategies that reconcile water savings with crop profitability in water-limited regions such as Middle Egypt.

Concurrently, nutrient management—especially potassium (K) fertilization—plays a crucial role in plant resilience under water stress. Potassium is vital for a range of physiological processes including enzyme activation, osmoregulation, stomatal regulation, and maintenance of turgor pressure; these functions collectively improve plant water-use efficiency and drought tolerance [10]. The use of K-bearing minerals, notably potassium-rich rocks such as feldspar (KAlSi_3_O_8_), as alternative or supplemental potassium sources has drawn renewed interest. Recent agronomic studies have demonstrated that feldspar, especially when combined with biological or organic amendments (e.g., humic acids, mycorrhizal fungi), can supply plant-available K and support crop growth and yield under certain soil conditions [11]. Such alternatives are particularly appealing in regions where chemical fertilizers are expensive or supply-limited, or where sustainable, low-cost fertilization strategies are needed.

Despite these advances, there remains a significant research gap: very few studies to date have addressed the combined (interactive) effects of irrigation regimes (e.g., extended intervals or deficit irrigation) and potassium source/type (chemical vs. natural mineral) on medicinal and aromatic plants such as fennel. Understanding this interaction is particularly important in arid and semi-arid regions like Middle Egypt, where water scarcity constrains both yield and quality of high-value essential-oil crops.

Therefore, the present study aims to evaluate the potential of feldspar as a partial or complete substitute for conventional potassium fertilizer (potassium sulfate) in fennel cultivation under Egyptian soil conditions, while also investigating how different irrigation intervals and potassium application rates influence fennel growth, yield, essential oil productivity, and water-use efficiency. In addition, the study seeks to determine whether potassium application—whether supplied from chemical or natural mineral sources—can enhance the plant’s tolerance to water deficit, thereby providing a sustainable agronomic strategy under limited water availability. Together, these objectives contribute to developing a more sustainable and cost-effective production system for fennel by integrating improved water management with efficient nutrient use, an approach that is increasingly important under the prevailing environmental and socio-economic pressures in Egypt. We hypothesized that (1) moderate deficit irrigation (extended intervals) would reduce vegetative growth and seed yield but potentially increase essential oil concentration due to stress-induced secondary metabolite accumulation, and (2) adequate potassium supply, particularly from highly soluble sources like potassium sulfate, would mitigate the adverse effects of water stress through improved osmoregulation, stomatal function, and nutrient uptake.

## 2. Results

### 2.1. Response of Soil Properties to Irrigation and Potassium Treatments

Across both seasons, potassium application significantly affected soil electrical conductivity (EC_e_) and organic matter compared with the control treatment (K0), whereas soil pH and bulk density did not show significant changes relative to K0 (Table 1). In 2022, EC_e_ increased significantly under K286 (feldspar), K572 (feldspar), K60 (K_2_SO_4_), and K120 (K_2_SO_4_) by approximately 6.6%, 10.7%, 0.0%, and 1.6%, respectively, compared with K0. In 2023, the corresponding increases in EC_e_ relative to K0 were about 5.6% for K286 (feldspar), 8.4% for K572 (feldspar), 3.5% for K60 (K_2_SO_4_), and 4.2% for K120 (K_2_SO_4_).

Soil organic matter content increased significantly under potassium treatments relative to the control in both seasons. In 2022, organic matter increased by approximately 4.1% under K286 (feldspar), 6.1% under K572 (feldspar), 4.8% under K60 (K_2_SO_4_), and 6.1% under K120 (K_2_SO_4_) compared with K0. In 2023, the increases relative to K0 were about 2.5% for K286 (feldspar), 4.3% for K572 (feldspar), 2.5% for K60 (K_2_SO_4_), and 4.3% for K120 (K_2_SO_4_).

Soil pH did not differ significantly among potassium treatments in either season, with changes relative to K0 remaining below 0.2%. Similarly, bulk density showed no significant variation among K treatments, with relative changes compared with K0 remaining within ±1% in both years.

Regarding irrigation intervals, extending the irrigation interval from 21 to 35 days resulted in a significant increase in EC_e_ compared with the shortest interval. Relative to the 21-day interval, EC_e_ increased by approximately 1.6% at 28 days and 4.8% at 35 days in 2022, and by about 2.1% and 4.8% in 2023, respectively. No significant percentage changes were detected in soil pH, organic matter, or bulk density among irrigation intervals in either season.

The interaction between irrigation interval and potassium treatment did not result in significant percentage changes relative to the control for any measured soil property in either growing season.

Across both growing seasons, soil-available nitrogen showed a significant response to irrigation interval but not to potassium treatments (Table 2). Compared with the 21-day irrigation interval, soil-available N decreased significantly by approximately 9.9% and 17.8% at 28 and 35 days, respectively, in 2022, and by about 9.7% and 18.8% in 2023. Relative to the control (K0), none of the potassium treatments resulted in significant percentage changes in soil-available N in either season, with variations remaining within ±4%.

Soil-available phosphorus did not differ significantly among irrigation intervals or potassium treatments in either season. Consequently, percentage changes in available P relative to the control (K0) remained below 3% across all potassium treatments and irrigation regimes in both years, with no significant deviations detected.

Soil-available potassium was significantly influenced by both irrigation interval and potassium fertilization. Relative to the 21-day irrigation interval, soil-available K decreased significantly by approximately 5.4% and 9.3% at 28 and 35 days, respectively, in 2022, and by about 8.6% and 12.7% in 2023. Compared with the control (K0), potassium treatments significantly increased soil-available K in both seasons. In 2022, soil-available K increased by approximately 13.1% under K286 (feldspar), 19.9% under K572 (feldspar), 8.0% under K60 (K_2_SO_4_), and 13.6% under K120 (K_2_SO_4_) relative to K0. In 2023, the corresponding increases relative to K0 were about 14.9%, 26.5%, 6.1%, and 16.6% for K286 (feldspar), K572 (feldspar), K60 (K_2_SO_4_), and K120 (K_2_SO_4_), respectively.

The interaction between irrigation interval and potassium treatment did not result in significant percentage changes relative to the control for soil-available nitrogen, phosphorus, or potassium in either season.

### 2.2. Response of Fennel Plants to Potassium Treatments Under Different Watering Regimes

Potassium fertilization significantly affected plant height in both growing seasons compared with the control (K0) (Table 3). In 2022, plant height increased by approximately 4.5% under K286 (feldspar), 10.2% under K572 (feldspar), 7.3% under K60 (K_2_SO_4_), and 11.7% under K4 relative to K0. In 2023, the corresponding increases compared with K0 were about 4.0%, 10.0%, 6.5%, and 10.9% under K286 (feldspar), K572 (feldspar), K60 (K_2_SO_4_), and K120 (K_2_SO_4_), respectively. Irrigation interval also significantly influenced plant height, with plants irrigated every 28 and 35 days showing significant reductions relative to the 21-day interval. Compared with the 21-day interval, plant height decreased by approximately 3.2% and 7.3% in 2022, and by about 2.6% and 7.6% in 2023, under 28- and 35-day irrigation intervals, respectively.

The number of branches per plant showed significant differences among potassium treatments in both seasons when compared with K0. In 2022, the number of branches increased by approximately 6.7% under K286 (feldspar), 14.4% under K572 (feldspar), 10.6% under K60 (K_2_SO_4_), and 16.1% under K120 (K_2_SO_4_) relative to the control. In 2023, increases relative to K0 were about 5.9%, 13.8%, 10.5%, and 16.1% under K286 (feldspar), K572 (feldspar), K60 (K_2_SO_4_), and K120 (K_2_SO_4_), respectively. Irrigation interval had a significant effect on branch number, with reductions of approximately 1.1% and 14.8% in 2022 and 1.9% and 15.4% in 2023 at 28 and 35 days, respectively, compared with the 21-day interval.

Stem diameter responded significantly to potassium treatments relative to K0 in both seasons. In 2022, stem diameter increased by approximately 13.8% under K286 (feldspar), 21.9% under K572 (feldspar), 16.9% under K60 (K_2_SO_4_), and 26.3% under K120 (K_2_SO_4_) compared with the control. In 2023, the corresponding increases relative to K0 were about 12.7%, 18.8%, 16.4%, and 21.2% under K286 (feldspar), K572 (feldspar), K60 (K_2_SO_4_), and K120 (K_2_SO_4_), respectively. Irrigation interval also significantly affected stem diameter, with reductions of approximately 1.6% and 8.9% in 2022 and 2.6% and 8.7% in 2023 at 28 and 35 days, respectively, compared with the 21-day interval.

The interaction between irrigation interval and potassium treatment did not result in significant percentage changes relative to the control for plant height, number of branches, or stem diameter in either growing season.

### 2.3. Response of Yield Components of Fennel Plants to Potassium Treatments Under Different Watering Regimes

Across irrigation intervals, potassium treatments produced significant percentage changes in all measured traits compared with the control (K0) (Table 4). Under the 21-day irrigation interval, the number of umbels per plant increased by approximately 34–52% in K286 (feldspar), 47–63% in K572 (feldspar), 38–55% in K60 (K_2_SO_4_), and 52–65% in K120 (K_2_SO_4_) relative to K0 across the two seasons. The number of umbellate per umbel showed increases of about 36–38% with K286 (feldspar), 49–53% with K572 (feldspar), 38–41% with K60 (K_2_SO_4_), and 50–54% with K120 (K_2_SO_4_) compared with the control. The 1000-seed weight increased by nearly 39–49% in K286 (feldspar), 64–65% in K572 (feldspar), 41–51% in K60 (K_2_SO_4_), and 69–80% in K120 (K_2_SO_4_) relative to K0.

Under the 28-day irrigation interval, potassium application resulted in marked percentage increases compared with K0. The number of umbels per plant increased by about 55–57% with K286 (feldspar), 75–78% with K572 (feldspar), 64–66% with K60 (K_2_SO_4_), and 81–82% with K120 (K_2_SO_4_) across seasons. The number of umbellate per umbel increased by approximately 38–36% in K286 (feldspar), 85–76% in K572 (feldspar), 41–40% in K60 (K_2_SO_4_), and 87–80% in K120 (K_2_SO_4_) relative to the control. The 1000-seed weight showed increases of about 63–68% with K286 (feldspar), 127–132% with K572 (feldspar), 68–73% with K60 (K_2_SO_4_), and 129–130% with K120 (K_2_SO_4_) compared with K0.

At the 35-day irrigation interval, all potassium treatments significantly increased measured traits relative to the control. The number of umbels per plant increased by approximately 55–46% in K286 (feldspar), 80–67% in K572 (feldspar), 62–46% in K60 (K_2_SO_4_), and 81–71% in K120 (K_2_SO_4_) across seasons. The number of umbellate per umbel increased by about 49–43% with K286 (feldspar), 82–75% with K572 (feldspar), 53–45% with K60 (K_2_SO_4_), and 86–77% with K120 (K_2_SO_4_) relative to K0. The 1000-seed weight increased by approximately 17–17% in K286 (feldspar), 119–96% in K572 (feldspar), 21–19% in K60 (K_2_SO_4_), and 123–98% in K120 (K_2_SO_4_) compared with the control treatment.

### 2.4. Response of Herb and Seed Yield of Fennel Plants to Potassium Treatments Under Different Watering Regimes

The results presented in Table 5 show that potassium treatments, expressed as percentage change relative to the control (K0), significantly affected all measured traits under the three irrigation intervals (21, 28, and 35 days). Across irrigation intervals, increases relative to K0 were calculated only where statistically significant differences occurred, and the magnitude of change is reported without restating absolute values.

For herb dry yield, all K treatments resulted in significant increases compared with K0 under the three irrigation intervals. Under the 21-day irrigation interval, K286 (feldspar), K572 (feldspar), K60 (K_2_SO_4_), and K120 (K_2_SO_4_) increased herb dry yield by approximately 17–21%, 20–23%, 18–20%, and 21–24%, respectively, compared with the control. Under the 28-day interval, the corresponding increases over K0 ranged from about 5–7% for K286 (feldspar), 25–27% for K572 (feldspar), 6–8% for K60 (K_2_SO_4_), and 26–28% for K120 (K_2_SO_4_). Under the 35-day interval, herb dry yield increased by roughly 36–38% with K286 (feldspar), 46–49% with K572 (feldspar), 37–39% with K60 (K_2_SO_4_), and 46–49% with K120 (K_2_SO_4_) compared with K0.

Seed yield exhibited marked percentage increases in response to K application relative to K0 across all irrigation intervals. Under the 21-day interval, K286 (feldspar) increased seed yield by about 50–57%, while K572 (feldspar) and K120 (K_2_SO_4_) resulted in increases of approximately 78–82%, and K60 (K_2_SO_4_) increased seed yield by about 54–57% compared with the control. Under the 28-day interval, seed yield increased by nearly 67–71% with K286 (feldspar), about 96–100% with K572 (feldspar), 71–75% with K60 (K_2_SO_4_), and 96–100% with K120 (K_2_SO_4_) relative to K0. Under the 35-day irrigation interval, increases over K0 were approximately 50–55% for K286 (feldspar) and K60 (K_2_SO_4_), and around 95–100% for K572 (feldspar) and K120 (K_2_SO_4_).

Biological yield also showed significant percentage increases relative to the control treatment. Under the 21-day irrigation interval, K286 (feldspar) increased biological yield by about 24–26%, K572 (feldspar) by approximately 31–33%, K60 (K_2_SO_4_) by about 0–2% (where significant), and K120 (K_2_SO_4_) by around 32–34% compared with K0. Under the 28-day interval, biological yield increased by roughly 16–18% with K286 (feldspar), 36–38% with K572 (feldspar), 16–18% with K60 (K_2_SO_4_), and 37–40% with K120 (K_2_SO_4_) relative to the control. Under the 35-day interval, increases over K0 were approximately 39–42% for K286 (feldspar), 56–58% for K572 (feldspar), 40–43% for K60 (K_2_SO_4_), and 57–59% for K120 (K_2_SO_4_).

Overall, the percentage change analysis relative to K0 clearly demonstrates the magnitude of response of herb dry yield, seed yield, and biological yield to different potassium treatments under varying irrigation intervals, as summarized in Table 5, without reiterating the numerical values reported in the table.

### 2.5. Response of Oil Yield of Fennel Plants to Potassium Treatments Under Different Watering Regimes

The results presented in Table 6 indicate that potassium application caused significant percentage changes in oil percentage and oil yield compared with the control treatment (K0) under the three irrigation intervals (21, 28, and 35 days). Only statistically significant differences are expressed as percentage changes relative to K0, and absolute numerical values from the table are not reiterated.

For oil percentage, all K treatments showed significant increases relative to the control across irrigation intervals. Under the 21-day irrigation interval, oil percentage increased by approximately 3–4% with K286 (feldspar), 5–6% with K572 (feldspar), 3–4% with K60 (K_2_SO_4_), and about 4–5% with K120 (K_2_SO_4_) compared with K0. Under the 28-day interval, the increase relative to the control ranged from about 2–3% for K286 (feldspar), 4–5% for K572 (feldspar), 3–4% for K60 (K_2_SO_4_), and 4–5% for K120 (K_2_SO_4_). Under the 35-day irrigation interval, oil percentage increased by roughly 2–3% with K286 (feldspar) and K60 (K_2_SO_4_), while K572 (feldspar) and K120 (K_2_SO_4_) resulted in increases of approximately 4–5% compared with the control.

Oil yield exhibited substantially larger percentage changes in response to potassium treatments compared with K0 under all irrigation intervals. Under the 21-day interval, oil yield increased by approximately 56–60% with K286 (feldspar), 89–92% with K572 (feldspar), 58–62% with K60 (K_2_SO_4_), and about 88–90% with K120 (K_2_SO_4_) relative to the control. Under the 28-day irrigation interval, the corresponding increases over K0 were around 70–75% for K286 (feldspar), 101–105% for K572 (feldspar), 72–76% for K60 (K_2_SO_4_), and 103–110% for K120 (K_2_SO_4_). Under the 35-day interval, oil yield increased by approximately 55–60% with K286 (feldspar) and K60 (K_2_SO_4_), while increases of about 105–110% were recorded with K572 (feldspar) and K120 (K_2_SO_4_) compared with the control.

Overall, expressing the results as percentage changes relative to K0 highlights the magnitude of response of oil percentage and oil yield to different potassium treatments under varying irrigation intervals, as summarized in Table 6, without restating the tabulated data or providing interpretation of the observed effects.

### 2.6. Response of NPK Content in Herb and Seeds of Fennel Plants to Potassium Treatments Under Different Watering Regimes

The results summarized in Table 7 show that potassium treatments, expressed as percentage change relative to the control (K0), significantly affected seed N, P, and K contents under the three irrigation intervals (21, 28, and 35 days). Only statistically significant differences are presented as percentage changes compared with K0, without repeating the absolute values reported in the table.

For seed nitrogen content, all K treatments resulted in significant increases relative to the control across irrigation intervals. Under the 21-day irrigation interval, seed N content increased by approximately 5–7% with K286 (feldspar), 13–15% with K572 (feldspar), 6–8% with K60 (K_2_SO_4_), and 15–17% with K120 (K_2_SO_4_) compared with K0. Under the 28-day interval, the corresponding increases over the control were about 12–14% for K286 (feldspar), 18–20% for K572 (feldspar), 13–15% for K60 (K_2_SO_4_), and 20–22% for K120 (K_2_SO_4_). Under the 35-day irrigation interval, seed N content increased by approximately 17–19% with K286 (feldspar), 33–35% with K572 (feldspar), 19–21% with K60 (K_2_SO_4_), and 34–36% with K120 (K_2_SO_4_) relative to K0.

Seed phosphorus content also showed significant percentage increases in response to potassium application. Under the 21-day interval, P content increased by about 19–21% with K286 (feldspar), 42–45% with K572 (feldspar), 27–30% with K60 (K_2_SO_4_), and 50–55% with K120 (K_2_SO_4_) compared with the control. Under the 28-day irrigation interval, increases over K0 were approximately 23–25% for K286 (feldspar), 41–45% for K572 (feldspar), 32–35% for K60 (K_2_SO_4_), and 50–55% for K120 (K_2_SO_4_). Under the 35-day interval, seed P content increased by roughly 17–20% with K286 (feldspar), 39–42% with K572 (feldspar), 22–25% with K60 (K_2_SO_4_), and 50–55% with K120 (K_2_SO_4_) compared with K0.

Seed potassium content exhibited smaller but significant percentage increases relative to the control. Under the 21-day irrigation interval, K286 (feldspar) increased seed K content by about 3–4%, K572 (feldspar) by 6–7%, K60 (K_2_SO_4_) by 4–5%, and K120 (K_2_SO_4_) by approximately 6–7% compared with K0. Under the 28-day interval, the corresponding increases were around 1–2% for K286 (feldspar), 4–5% for K572 (feldspar), 1–2% for K60 (K_2_SO_4_), and 4–5% for K120 (K_2_SO_4_). Under the 35-day irrigation interval, seed K content increased by approximately 4–5% with K286 (feldspar), 7–8% with K572 (feldspar), 5–6% with K60 (K_2_SO_4_), and 7–8% with K120 (K_2_SO_4_) relative to the control.

Overall, presenting the results as percentage changes relative to K0 highlights the magnitude of variation in seed N, P, and K contents associated with potassium treatments under different irrigation intervals, as shown in Table 7, without reiterating tabulated values or providing interpretation of the observed responses

Across potassium treatments, all measured traits were expressed as percentage change relative to the control (K0) (Table 8). For N content in herb, K286 (feldspar) increased N by 2.9% and 3.6% in 2022 and 2023, respectively, compared with K0, while K572 (feldspar) increased N by 8.6% and 8.8%. K60 (K_2_SO_4_) showed increases of 4.3% in 2022 and 4.4% in 2023, and K120 (K_2_SO_4_) increased N by 10.7% in 2022 and 13.1% in 2023. For P content, K286 (feldspar) increased P by 25.0% in 2022 and 21.7% in 2023 relative to K0, whereas K572 (feldspar) increased P by 37.5% and 34.8% in the respective seasons. K60 (K_2_SO_4_) showed increases of 29.2% in 2022 and 21.7% in 2023, while K120 (K_2_SO_4_) resulted in increases of 45.8% in 2022 and 39.1% in 2023. For K content in herb, K286 (feldspar) increased values by 2.3% in 2022 and 2.4% in 2023 relative to K0, K572 (feldspar) by 4.2% and 4.3%, K60 (K_2_SO_4_) by 2.8% and 2.8%, and K120 (K_2_SO_4_) by 4.7% and 4.7% in the two seasons, respectively.

When irrigation intervals were compared with the 21-day interval as the reference, N content at 28 days decreased by 5.2% in 2022 and 5.3% in 2023, while at 35 days it decreased by 7.8% and 8.6%, respectively. P content decreased by 6.1% in 2022 and 9.7% in 2023 at 28 days, and by 15.2% and 12.9% at 35 days, relative to the 21-day interval. K content decreased by 1.4% in both seasons at 28 days and by 2.7% in 2022 and 2.7% in 2023 at 35 days, compared with the 21-day interval.

For the interaction between irrigation interval and potassium treatments, percentage changes were calculated relative to the corresponding K0 within each irrigation regime. Under the 21-day interval, N content increased by 3.5–14.7% in 2022 and by 4.3–19.3% in 2023 across K286 (feldspar)–K120 (K_2_SO_4_) compared with K0, while P increased by 22.2–37.0% in 2022 and by 11.1–29.6% in 2023, and K increased by 2.3–4.6% in 2022 and by 2.3–4.7% in 2023. Under the 28-day interval, N increased by 3.6–10.1% in 2022 and by 3.7–13.2% in 2023 relative to K0, P increased by 20.8–45.8% in 2022 and by 17.4–34.8% in 2023, and K increased by 2.3–4.7% in 2022 and by 2.4–4.3% in 2023. Under the 35-day interval, N increased by 1.5–7.3% in 2022 and by 3.0–7.5% in 2023 relative to K0, P increased by 28.6–52.4% in 2022 and by 30.0–55.0% in 2023, and K increased by 2.9–4.8% in 2022 and by 2.4–5.3% in 2023.

### 2.7. Pearson Correlation Analysis Among Soil Properties, Growth, Yield, and Nutrient Traits

**Figure 1:**
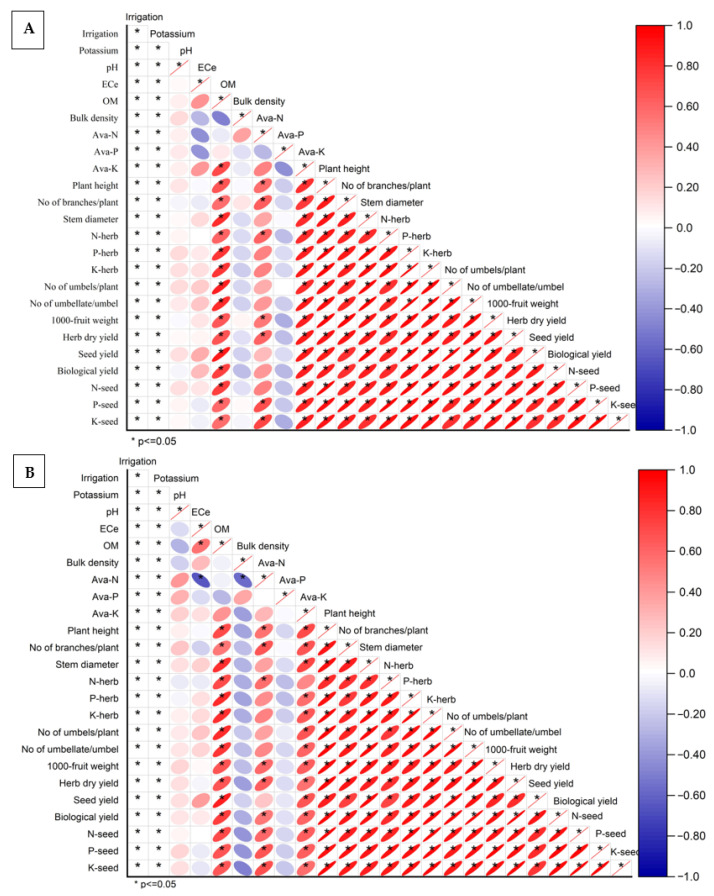
Pearson correlation matrices showing the relationships among irrigation regime, potassium treatments, soil properties (pH, EC_e_, organic matter, bulk density, and available N, P, and K), vegetative growth traits, yield components, yield parameters, and herb and seed nutrient contents in fennel during the 2022 (**A**) and 2023 (**B**) growing seasons. Red and blue colors indicate positive and negative correlations, respectively, with color intensity representing the strength of the correlation. Asterisks (*) indicate significant correlations at *p* ≤ 0.05.

The Pearson correlation analysis for the 2022 season showed that irrigation and potassium treatments were significantly correlated (*p* ≤ 0.05) with most soil, growth, yield, and nutrient traits (Figure 1A). Soil chemical and physical properties exhibited clear associations with plant performance. Electrical conductivity (ECe) was positively correlated with organic matter and available potassium, while bulk density showed negative correlations with available nutrients and most growth traits. Available N, P, and K were positively correlated with plant height, number of branches, stem diameter, and herb nutrient contents (N-herb, P-herb, and K-herb). Strong positive correlations were also observed among vegetative growth traits themselves, indicating close relationships between plant height, branching, stem thickness, and nutrient accumulation in the herb. Yield components, including number of umbels per plant, number of umbellates per umbel, 1000-fruit weight, herb dry yield, seed yield, and biological yield, were highly and positively correlated with growth traits and herb nutrient contents. Seed nutrient contents (N-seed, P-seed, and K-seed) were likewise strongly correlated with seed yield and biological yield, reflecting consistent relationships between productivity and nutrient accumulation. Negative correlations were mainly associated with bulk density and, to a lesser extent, soil pH, which tended to be inversely related to available nutrients and several growth and yield attributes.

A similar correlation structure was observed in the 2023 season, confirming the stability of the relationships across years (Figure 1B). Irrigation and potassium treatments again showed significant correlations with most measured variables. Available soil nutrients maintained positive associations with vegetative growth traits, herb nutrient contents, and yield components. Plant height, number of branches, and stem diameter were strongly interrelated and positively correlated with yield traits such as herb dry yield, seed yield, biological yield, and yield components. Herb nutrient contents were positively correlated with both growth and yield variables, and seed nutrient contents showed strong positive relationships with seed yield and biological yield. As in 2022, bulk density exhibited negative correlations with available nutrients and several plant performance traits, while ECe and organic matter were positively associated with nutrient availability and plant growth. Overall, the correlation matrices for both seasons demonstrate consistent and significant interrelationships among soil properties, nutrient status, vegetative growth, yield components, and final yields under the different irrigation intervals and potassium treatments.

### 2.8. Principal Component Analysis of Soil and Plant Traits Under Different Irrigation Intervals and Potassium Treatments

**Figure 2:**
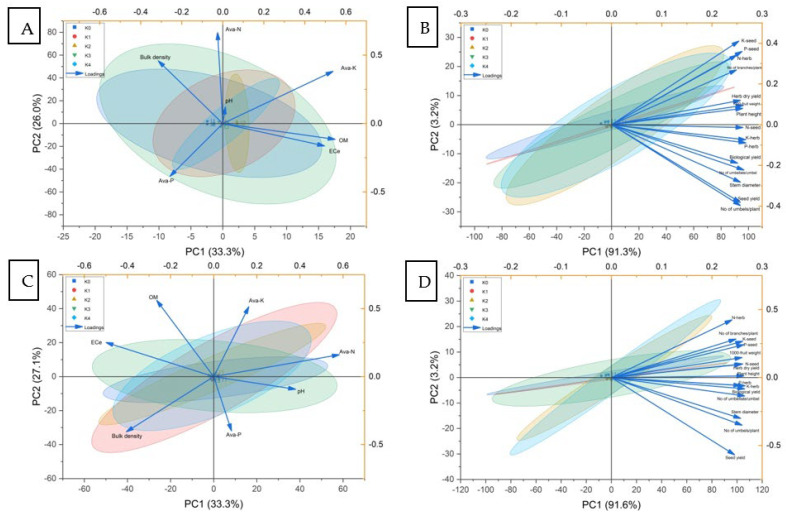
Principal component analysis (PCA) biplots illustrating the multivariate relationships among soil and plant traits under five potassium treatments (K0–K4) and three irrigation intervals (21, 28, and 35 days) during the 2022 and 2023 growing seasons: (**A**) soil traits 2022, (**B**) plant traits 2022, (**C**) soil traits 2023, and (**D**) plant traits 2023. Vectors represent variable loadings, and points represent treatment scores.

Principal component analysis (PCA) was applied to summarize the multivariate relationships among soil and plant traits in response to the five potassium treatments under the three irrigation intervals (21, 28, and 35 days) for the 2022 and 2023 seasons (Figure 2). For soil traits in 2022 (Figure 2A), the first two principal components accounted for most of the total variance and separated the treatments mainly according to gradients associated with nutrient availability and soil physical properties. Variables related to available nutrients (available N, P, and K), organic matter, and electrical conductivity loaded strongly in the same direction on the first principal component, whereas bulk density and soil pH were positioned in the opposite direction, indicating contrasting contributions to the overall variance structure. Treatment points were distributed along these axes, reflecting differences among irrigation intervals and potassium treatments in soil chemical and physical attributes.

The PCA of plant traits for 2022 (Figure 2B) showed that vegetative growth variables (plant height, number of branches, and stem diameter), yield components (number of umbels per plant, number of umbellates per umbel, and 1000-fruit weight), final yields (herb dry yield, seed yield, and biological yield), and nutrient contents in herb and seed (N, P, and K) were grouped closely and aligned with the positive side of the first principal component. This indicates that these variables contributed similarly to the overall variability among treatments. Treatments receiving higher potassium levels and shorter irrigation intervals tended to cluster in the same region of the biplot, whereas the control and longer irrigation interval treatments were positioned toward the opposite side, reflecting contrasting multivariate responses.

A comparable pattern was observed for soil traits in 2023 (Figure 2C), where the first two principal components again captured the major portion of variability. Available nutrients and organic matter showed positive loadings on the dominant axis, while bulk density and pH were oriented oppositely, indicating consistent soil trait relationships across seasons. The spatial distribution of treatment scores demonstrated a clear separation among irrigation intervals and potassium treatments, with similar grouping tendencies to those observed in 2022.

For plant traits in 2023 (Figure 2D), growth traits, yield attributes, and nutrient contents remained closely associated and loaded strongly on the first principal component, confirming the stability of trait associations between seasons. Yield-related variables and seed nutrient contents showed strong alignment, whereas treatments under different irrigation intervals and potassium levels were distributed along the principal axes, illustrating their combined influence on the multivariate plant response. Overall, the PCA results for both years demonstrate consistent clustering patterns and stable associations among soil properties, plant growth, yield components, and nutrient traits under varying irrigation regimes and potassium treatments.

## 3. Discussion

### 3.1. Influence of Irrigation Intervals on Soil Properties and Nutrient Availability

Irrigation scheduling significantly altered soil chemical properties, particularly EC_e_ and available potassium, whereas soil pH, organic matter content, and bulk density remained largely unaffected. The increase in soil EC_e_ with prolonged irrigation intervals (35 days) can be attributed to reduced leaching of soluble salts and higher evaporative concentration under arid climatic conditions. Similar trends have been widely reported in irrigated soils of arid and semi-arid regions, where extended drying cycles promote salt accumulation in the root zone due to upward capillary movement and limited downward water flux [9,12].

The observed decline in soil-available potassium under longer irrigation intervals highlights the strong dependency of potassium mobility on soil moisture. In conditions of reduced soil moisture, potassium also undergoes non-exchangeable sorption (fixation). Unlike nitrate, potassium moves predominantly by diffusion, a process that slows markedly as soil water content decreases [13,14,15]. Reduced soil moisture restricts K diffusion toward the rhizosphere and limits root uptake, even when total soil potassium reserves are adequate. This mechanism explains the progressive reduction in available K observed as irrigation intervals extended from 21 to 35 days, consistent with findings reported for several field crops under deficit irrigation [16].

Potassium fertilization significantly increased soil-available K after harvest, with potassium sulfate showing superior efficiency compared to feldspar. This difference reflects the higher solubility and immediate availability of potassium sulfate, whereas feldspar releases potassium slowly through weathering processes. Rock-based potassium sources, including feldspar, are known to contribute to soil K pools over time, but their short-term efficiency depends on soil texture, moisture, microbial activity, and root-induced mineral dissolution [17].

### 3.2. Effects of Irrigation Intervals on Vegetative Growth and Reproductive Development of Fennel

The significant reduction in plant height, branch number, and stem diameter under prolonged irrigation intervals reflects the sensitivity of fennel to water deficit during vegetative growth. Water stress limits cell expansion, reduces leaf area development, and accelerates leaf senescence, thereby shortening the effective photosynthetic period [18]. These constraints ultimately reduce assimilate availability for reproductive growth, as clearly evidenced by the reduced number of umbels and umbellates per plant under the 35-day irrigation interval.

Fennel, like many Apiaceae species, exhibits particular sensitivity to water stress during flowering and seed-filling stages. Previous studies have shown that drought stress during these stages increases floral abortion and reduces seed set, primarily due to impaired pollen viability and reduced carbohydrate supply to developing reproductive organs [19].

The pronounced decline in 1000-seed weight under extended irrigation intervals further indicates restricted assimilate translocation to seeds. Seed filling is highly dependent on sustained photosynthetic activity and efficient phloem transport, both of which are compromised under water-limited conditions.

### 3.3. Essential Oil Content and Yield Responses to Irrigation Regimes

Essential oil percentage exhibited a slight increase under prolonged irrigation intervals, while oil yield per hectare declined substantially. This contrasting response is commonly reported in medicinal and aromatic plants and is largely attributed to a concentration effect rather than enhanced biosynthesis [20]. Reduced seed biomass under water stress leads to higher oil concentration on a dry-weight basis, but the overall oil yield per unit area declines due to reduced fruit yield. In fennel, essential oil accumulation is closely linked to fruit development and carbon availability. Adequate water supply supports prolonged photosynthesis and assimilate translocation to fruits, thereby enhancing total oil yield despite relatively stable oil percentages [8].

### 3.4. Role of Potassium Fertilization in Enhancing Growth, Yield, and Nutrient Uptake

Potassium fertilization significantly improved vegetative growth, yield components, seed yield, and essential oil productivity across all irrigation regimes. The superior performance of potassium sulfate compared with feldspar reflects its immediate solubility and rapid uptake by plants. Potassium is indispensable for enzyme activation, protein synthesis, photosynthesis, and carbohydrate metabolism, processes that collectively support biomass production and reproductive development [10]. The enhancement of umbels per plant and seed weight under potassium fertilization indicates improved sink strength and assimilate partitioning. Potassium plays a central role in phloem loading and translocation of sugars from source leaves to developing seeds, thereby promoting efficient seed filling [21]. Feldspar application, despite its lower efficiency, significantly improved fennel performance relative to the unfertilized control. This confirms earlier findings that rock potassium sources can partially substitute soluble fertilizers, particularly under conditions of adequate soil moisture that facilitate mineral weathering [22].

### 3.5. Physiological Mechanisms Underlying Potassium-Mediated Mitigation of Water Stress

The significant irrigation × potassium interaction observed in this study highlights potassium’s crucial role in enhancing fennel tolerance to water deficit. Several well-established physiological mechanisms explain this response. Potassium is the dominant inorganic osmoticum in plant cells and is essential for maintaining cell turgor under fluctuating soil moisture conditions. Adequate potassium supply enhances osmotic adjustment, allowing cells to retain water and sustain growth under moderate drought stress [23]. This mechanism explains why potassium-fertilized plants under 28-day irrigation intervals maintained growth and yield comparable to more frequently irrigated plants. Guard cell turgor, and thus stomatal aperture, is directly regulated by potassium fluxes. Sufficient potassium enhances stomatal responsiveness, enabling plants to optimize CO_2_ uptake while minimizing transpirational water loss [24]. Improved stomatal control increases intrinsic water-use efficiency, particularly under intermittent water stress. Potassium activates numerous enzymes involved in photosynthesis and respiration and stabilizes chloroplast structure. Under water stress, potassium-deficient plants experience rapid declines in photosynthetic rate due to impaired ATP synthesis and Rubisco activity [25]. Adequate potassium supply therefore sustains photosynthetic carbon assimilation under moderate drought conditions. Potassium is essential for phloem transport of photoassimilates. Under potassium sufficiency, carbohydrate movement from leaves to developing umbels and seeds is enhanced, supporting reproductive resilience under water-limited conditions. This mechanism explains the higher seed weight and oil yield observed in potassium-fertilized treatments under extended irrigation intervals [26]. Essential oil biosynthesis depends on carbon skeleton availability and metabolic energy rather than direct potassium involvement. By sustaining photosynthesis, enzyme activity, and assimilate transport, potassium indirectly supports oil biosynthesis and accumulation in fennel fruits, particularly under moderate water stress [27].

### 3.6. Agronomic and Sustainability Implications

The results clearly demonstrate that optimized potassium nutrition can partially compensate for reduced irrigation frequency in fennel cultivation. While frequent irrigation (21 days) combined with potassium sulfate maximized productivity, extending irrigation to 28 days with adequate potassium supply produced comparable yields, offering substantial water savings. Feldspar, despite lower efficiency, represents a promising supplementary potassium source where fertilizer costs or availability constrain conventional fertilization. High-rate feldspar (572 kg ha^−1^) improved fennel performance relative to the unfertilized control but remained inferior to equivalent potassium sulfate rates (typically 15–25% lower in seed and oil yield), suggesting it may serve as a supplementary rather than complete replacement source, particularly in low-input systems. From an economic perspective, extending irrigation to 28 days saved approximately 23% of irrigation water while maintaining 85–90% of seed and oil yields (when combined with adequate potassium), potentially reducing pumping costs significantly in water-scarce regions. Although feldspar is cheaper and locally available compared to imported potassium sulfate, its lower short-term efficiency may require higher application rates or co-amendments (e.g., organic matter) to achieve comparable economic returns.

## 4. Materials and Methods

### 4.1. Experimental Site and Soil Characteristics

Field experiments were carried out during two consecutive growing seasons (2021/2022 and 2022/2023) at the Experimental Farm of the Horticultural Research Station, Agricultural Research Center (ARC), Beni-Suef Governorate, Egypt (29°24′ N; 31°04′ E; 30–40 m above sea level). Meteorological data are presented in Table 9. Before planting, representative soil samples (0–30 cm) were collected and analyzed according to Association of Official Analytical Chemists (AOAC) [28]. The soil was clay in texture (clay 51.16%, silt 30.37%, and sand 18.47%) with pH values (measured in 1:2.5 soil:distilled water suspension) of 8.0 and 8.1, EC_e_ values of 1.22 and 1.47 dS m^−1^, organic matter contents of 15 and 16 g kg^−1^, bulk density levels of 1.22 and 1.70 g cm^−3^, available water values of 21.56 and 21.54%, wilting point values of 20.30 and 20.11%, and available nutrients including nitrogen (35.1 and 32.2 mg kg^−1^), phosphorus (15.7 and 17.1 mg kg^−1^), and potassium (196 and 202 mg kg^−1^) in the first and second seasons, respectively. According to the IUSS Working Group WRB [29], the soil at the experimental site is classified as a Vertisol.

### 4.2. Experimental Design and Treatments

The experiment was conducted in an arid region characterized by extremely low and irregular rainfall. During the experimental seasons, no effective rainfall events occurred that could interfere with the imposed irrigation treatments. Therefore, the irrigation treatments were applied under natural open-field conditions without the need for rain shelters, as precipitation did not contribute to soil moisture during the study period. The experiment followed a split-plot design with four replications. Irrigation intervals were assigned to the main plots and consisted of three regimes. In the first regime (I_1_), irrigation was applied every 21 days, including five irrigations in addition to the planting irrigation, with a total applied water amount of approximately 7426 m^3^ ha^−1^. The second regime (I_2_) involved irrigation every 28 days, including four irrigations in addition to the planting irrigation, with a total applied water amount of approximately 5736 m^3^ ha^−1^. The third regime (I_3_) consisted of irrigation every 35 days, including three irrigations in addition to the planting irrigation, with a total applied water amount of approximately 4379 m^3^ ha^−1^. The EC of the irrigation water used in all regimes was 0.44 dS m^−1^, and the pH value was 7.13. All irrigation treatments received an initial irrigation immediately after sowing. Irrigation intervals were designed to represent full irrigation (21 days), moderate deficit (28 days), and severe deficit (35 days). Scheduling was based on replenishing soil water depletion to field capacity after reaching approximately 50% allowable depletion in the root zone (0–60 cm) for the 21-day interval, with longer intervals allowing greater depletion before refilling. Total applied water amounts (approximately 7426, 5736, and 4379 m^3^ ha^−1^ for 21-, 28-, and 35-day intervals, respectively) were calculated from measured soil moisture deficits and reference evapotranspiration data obtained from a local meteorological station.

Potassium fertilization treatments were allocated to the subplots and included five levels: K_0_ (control, no potassium), K286 (50% recommended feldspar rate providing 29 kg K_2_O ha^−1^ about 286 kg feldspar), K572 (100% recommended feldspar rate providing 57 kg K_2_O ha^−1^ about 572 kg feldspar), K60 (50% recommended potassium sulfate providing 29 kg K_2_O ha^−1^ about 60 kg K_2_SO_4_), and K120 (100% recommended potassium sulfate providing 57 kg K_2_O ha^−1^ about 120 kg K_2_SO_4_). The feldspar used was a local K-feldspar source containing approximately 10% K_2_O, finely ground to pass through a 2-mm sieve (particle size < 2 mm) to enhance dissolution. Feldspar treatments were applied during land preparation, while potassium sulfate was applied in two equal doses at 30 and 60 days after planting. Nitrogen fertilizer was applied as ammonium nitrate (35.5% N) at 143 kg N ha^−1^ in two equal splits after thinning (30 and 60 days), and phosphorus fertilizer was added at 120 kg ha^−1^ as calcium superphosphate (15.5% P_2_O_5_) during soil preparation before planting.

### 4.3. Crop Establishment and Field Management

Each experimental plot measured 3 × 3.5 m (10.5 m^2^) and consisted of five ridges spaced 0.6 m apart. Fennel seeds obtained from the Medicinal and Aromatic Plants Department, Agricultural Research Center, Egypt, were sown on October 10 in the first season and October 20 in the second season at a spacing of 0.4 m along ridge sides. Plants were thinned to two plants per hill 30 days after emergence. All recommended agricultural practices—including hoeing, weed control, pest and disease management, and routine field operations—were uniformly applied throughout the experiment to ensure standard crop management.

### 4.4. Soil and Plant Chemical Analysis

At crop maturity, soil material was obtained from the surface layer (0–30 cm). The collected samples were placed in polyethylene containers, allowed to dry under ambient laboratory conditions, and then passed through a 2-mm sieve prior to analysis. Soil reaction was assessed in an aqueous suspension prepared at a soil-to-water ratio of 1:2.5 using a digital pH meter (Genway 3510, Cole-Parmer, Cambridgeshire, UK) [30]. Salinity was evaluated by measuring EC_e_ in a saturated soil paste extract at 25 °C with an EC meter (Jenway 4310, Cole-Parmer, Cambridgeshire, UK) [30]. Organic matter content was estimated following the Walkley–Black wet oxidation procedure [30]. Mineral nitrogen availability was determined after extraction with 2 M KCl, followed by quantification using the Kjeldahl digestion method [30]. Plant-available phosphorus was extracted using 0.5 M sodium bicarbonate according to the Olsen procedure [31] and measured colorimetrically with a spectrophotometer (UV-160A spectrophotometer; Shimadzu, Kyoto, Japan) [32]. Exchangeable potassium was obtained by extraction with 1 M ammonium acetate and analyzed using a flame photometer (Sherwood Model 410, Sherwood Scientific Ltd., Cambridgeshire, UK) [33]. Soil bulk density was measured using intact core samples, and total porosity was subsequently calculated based on bulk and particle density values [12].

Nitrogen, phosphorus, and potassium concentrations in herb and seed tissues were determined following acid digestion of plant material. Briefly, 0.2 g of finely powdered sample was transferred into a Kjeldahl digestion vessel, to which 5 mL of concentrated sulfuric acid (95–97% H_2_SO_4_; density 1.84 kg L^−1^; Merck, Darmstadt Hessen, Germany) was added. The digestion process was initiated by heating the tubes with a controlled temperature increase of 5 °C min^−1^ until 170 °C was reached, and this temperature was maintained for two hours. After allowing the digests to cool for approximately 30 min, 2 mL of perchloric acid (30% HClO_4_) was introduced, followed by further heating at 120 °C for one additional hour until complete clarification of the solution occurred. The resulting digest was then diluted to a final volume of 50 mL using ultrapure water. Total nitrogen was quantified using the Kjeldahl procedure [30], whereas phosphorus and potassium concentrations were determined by atomic absorption spectrophotometry (PerkinElmer 3300, PerkinElmer, Inc., Shelton, CT, USA), with an analytical detection limit of 100 ppb [28].

### 4.5. Growth, Yield, and Essential Oil Measurements

At harvest, five plants were randomly sampled from each plot to evaluate vegetative growth traits including plant height, number of branches per plant, stem diameter, and dry herb weight. Yield components were recorded by determining the number of umbels per plant, number of umbellates per umbel, and 1000-seed weight. Yield measurements included herb yield per plant, herb yield per hectare, fruit yield per plant, fruit yield per hectare, and total biological yield. Essential oil percentage was determined from dried fruits using hydrodistillation with a Clevenger apparatus, and oil yield was calculated on both a per-plant and per-hectare basis, following the AOAC [28] method.

### 4.6. Statistical Analysis

All collected data were subjected to statistical analysis using a split-plot ANOVA design. Normality of residuals and homogeneity of variance were verified using Shapiro–Wilk and Levene’s tests, respectively; no data transformation was required as assumptions were met. All statistical analyses were conducted using IBM SPSS Statistics v.27.0 (IBM Corp., Armonk, NY, USA). Treatment means were compared using the least significant difference (LSD) test at the 5% probability level. Statistical analyses were performed using SPSS.

## 5. Conclusions

The present study demonstrates that fennel productivity under arid conditions is influenced by the interaction between irrigation scheduling and potassium nutrition. Frequent irrigation at 21-day intervals supported higher vegetative growth, yield components, seed yield, and essential oil yield, while extending irrigation to 28 days maintained acceptable yield levels when sufficient potassium was supplied. Potassium sulfate was more effective than feldspar in improving growth, nutrient uptake, and oil yield. However, high application rates of feldspar also enhanced fennel performance compared with the unfertilized control, indicating its potential as an alternative potassium source. Future studies should focus on multi-location and multi-year evaluations to assess the consistency of these responses and their long-term effects on soil potassium dynamics. Additional work may examine strategies to improve the efficiency of rock-based potassium sources and to further characterize crop physiological responses and economic feasibility under different irrigation and potassium management regimes.

## Figures and Tables

**Table 1:** Effects of irrigation intervals (21, 28, and 35 days) and different potassium levels and sources (potassium sulfate or feldspar; kg ha^−1^) on selected soil properties under fennel (*Foeniculum vulgare* Mill.) cultivation.

Treatments		pH		EC_e_ (dS m^−1^)		Organic Matter (g kg^−1^)		Bulk Density (g cm^−3^)	
		2022	2023	2022	2023	2022	2023	2022	2023
Irrigation intervals	21 days	8.02 ± 0.02 a	8.14 ± 0.02 a	1.24 ± 0.02 c	1.46 ± 0.01 c	15.3 ± 0.4 a	16.7 ± 0.2 a	1.23 ± 0.01 a	1.21 ± 0.01 a
	28 days	8.01 ± 0.02 a	8.14 ± 0.02 a	1.26 ± 0.01 b	1.49 ± 0.01 b	15.3 ± 0.3 a	16.6 ± 0.3 a	1.22 ± 0.01 a	1.22 ± 0.01 a
	35 days	8.02 ± 0.02 a	8.13 ± 0.01 a	1.30 ± 0.01 a	1.53 ± 0.01 a	15.3 ± 0.3 a	16.7 ± 0.2 a	1.22 ± 0.02 a	1.22 ± 0.02 a
K levels and sources	K_0_	8.01 ± 0.01 a	8.14 ± 0.01 a	1.22 ± 0.00 c	1.43 ± 0.03 b	14.7 ± 0.4 c	16.2 ± 0.1 c	1.23 ± 0.02 a	1.21 ± 0.01 a
	K286 (feldspar)	8.02 ± 0.02 a	8.15 ± 0.02 a	1.30 ± 0.02 b	1.51 ± 0.02 a	15.3 ± 0.2 b	16.6 ± 0.4 b	1.23 ± 0.02 a	1.21 ± 0.01 a
	K572 (feldspar)	8.01 ± 0.01 a	8.14 ± 0.02 a	1.35 ± 0.03 a	1.55 ± 0.01 a	15.6 ± 0.5 a	16.9 ± 0.5 a	1.22 ± 0.01 a	1.21 ± 0.02 a
	K60 (K_2_SO_4_)	8.02 ± 0.01 a	8.14 ± 0.01 a	1.22 ± 0.02 c	1.48 ± 0.02 b	15.4 ± 0.3 b	16.6 ± 0.3 b	1.22 ± 0.01 a	1.21 ± 0.01 a
	K120 (K_2_SO_4_)	8.01 ± 0.01 a	8.13 ± 0.01 a	1.24 ± 0.01 c	1.49 ± 0.02 b	15.6 ± 0.4 a	16.9 ± 0.3 a	1.22 ± 0.02 a	1.22 ± 0.01 a
Interactions									
21 days	K_0_	8.02 ± 0.02 a	8.14 ± 0.02 a	1.19 ± 0.00 a	1.39 ± 0.03 a	14.6 ± 0.4 a	16.2 ± 0.4 a	1.23 ± 0.01 a	1.21 ± 0.02 a
	K286 (feldspar)	8.01 ± 0.02 a	8.15 ± 0.02 a	1.28 ± 0.01 a	1.48 ± 0.01 a	15.3 ± 0.6 a	16.6 ± 0.3 a	1.23 ± 0.02 a	1.20 ± 0.02 a
	K572 (feldspar)	8.02 ± 0.01 a	8.14 ± 0.01 a	1.32 ± 0.01 a	1.52 ± 0.02 a	15.5 ± 0.5 a	16.9 ± 0.3 a	1.22 ± 0.01 a	1.20 ± 0.01 a
	K60 (K_2_SO_4_)	8.02 ± 0.01 a	8.16 ± 0.01 a	1.20 ± 0.02 a	1.46 ± 0.02 a	15.4 ± 0.3 a	16.6 ± 0.2 a	1.23 ± 0.02 a	1.21 ± 0.01 a
	K120 (K_2_SO_4_)	8.01 ± 0.01 a	8.13 ± 0.00 a	1.22 ± 0.02 a	1.45 ± 0.01 a	15.6 ± 0.3 a	17.0 ± 0.2 a	1.22 ± 0.02 a	1.21 ± 0.01 a
28 days	K_0_	8.00 ± 0.01 a	8.13 ± 0.01 a	1.21 ± 0.01 a	1.42 ± 0.02 a	14.9 ± 0.3 a	16.3 ± 0.2 a	1.22 ± 0.01 a	1.21 ± 0.02 a
	K286 (feldspar)	8.01 ± 0.02 a	8.15 ± 0.02 a	1.30 ± 0.02 a	1.51 ± 0.02 a	15.3 ± 0.1 a	16.6 ± 0.2 a	1.23 ± 0.01 a	1.22 ± 0.02 a
	K572 (feldspar)	8.01 ± 0.02 a	8.14 ± 0.02 a	1.35 ± 0.02 a	1.55 ± 0.03 a	15.6 ± 0.2 a	16.8 ± 0.3 a	1.22 ± 0.02 a	1.22 ± 0.02 a
	K60 (K_2_SO_4_)	8.02 ± 0.02 a	8.14 ± 0.01 a	1.22 ± 0.02 a	1.48 ± 0.01 a	15.3 ± 0.2 a	16.6 ± 0.3 a	1.23 ± 0.01 a	1.21 ± 0.01 a
	K120 (K_2_SO_4_)	8.01 ± 0.01 a	8.13 ± 0.01 a	1.24 ± 0.00 a	1.48 ± 0.02 a	15.6 ± 0.5 a	16.9 ± 0.3 a	1.22 ± 0.01 a	1.22 ± 0.01 a
35 days	K_0_	8.01 ± 0.02 a	8.14 ± 0.00 a	1.26 ± 0.01 a	1.49 ± 0.03 a	14.6 ± 0.3 a	16.2 ± 0.3 a	1.23 ± 0.02 a	1.22 ± 0.02 a
	K286 (feldspar)	8.03 ± 0.01 a	8.14 ± 0.01 a	1.32 ± 0.02 a	1.55 ± 0.02 a	15.3 ± 0.3 a	16.6 ± 0.2 a	1.22 ± 0.01 a	1.22 ± 0.01 a
	K572 (feldspar)	8.01 ± 0.01 a	8.13 ± 0.02 a	1.39 ± 0.02 a	1.59 ± 0.02 a	15.6 ± 0.4 a	16.9 ± 0.2 a	1.22 ± 0.02 a	1.21 ± 0.02 a
	K60 (K_2_SO_4_)	8.01 ± 0.02 a	8.13 ± 0.02 a	1.25 ± 0.01 a	1.51 ± 0.01 a	15.4 ± 0.5 a	16.7 ± 0.2 a	1.21 ± 0.01 a	1.21 ± 0.02 a
	K120 (K_2_SO_4_)	8.02 ± 0.01 a	8.13 ± 0.02 a	1.26 ± 0.01 a	1.53 ± 0.03 a	15.6 ± 0.3 a	16.9 ± 0.3 a	1.22 ± 0.01 a	1.22 ± 0.01 a

K_0_: 0.0 kg K_2_O ha^−1^. Means followed by different letters are significant at 0.05 level of LSD. Data are means ± SD. n = 3.

**Table 2:** Effects of irrigation intervals (21, 28, and 35 days) and different potassium levels and sources (potassium sulfate or feldspar; kg ha^−1^) on soil fertility under fennel (*Foeniculum vulgare* Mill.) cultivation.

Treatments		Soil-Available N (mg kg^−1^)		Soil-Available P (mg kg^−1^)		Soil-Available K (mg kg^−1^)	
		2022	2023	2022	2023	2022	2023
Irrigation intervals	21 days	35.4 ± 0.7 a	32.0 ± 0.8 a	15.2 ± 0.3 a	17.2 ± 0.5 a	205 ± 14 a	220 ± 15 a
	28 days	31.9 ± 0.8 b	28.9 ± 0.9 b	15.3 ± 0.3 a	17.5 ± 0.4 a	194 ± 14 b	201 ± 14 b
	35 days	29.1 ± 0.9 c	26.0 ± 0.4 c	15.4 ± 0.3 a	17.2 ± 0.3 a	186 ± 16 c	192 ± 16 c
K levels and sources	K_0_	31.1 ± 0.7 a	29.3 ± 0.6 a	15.2 ± 0.2 a	17.2 ± 0.3 a	176 ± 15 d	181 ± 15 d
	K286 (feldspar)	31.9 ± 0.8 a	29.1 ± 0.5 a	15.3 ± 0.1 a	17.4 ± 0.2 a	199 ± 11 b	208 ± 13 b
	K572 (feldspar)	31.8 ± 0.9 a	28.7 ± 0.7 a	15.0 ± 0.4 a	17.0 ± 0.5 a	211 ± 13 a	229 ± 11 a
	K60 (K_2_SO_4_)	32.4 ± 1.0 a	28.5 ± 0.8 a	15.6 ± 0.3 a	17.1 ± 0.4 a	190 ± 14 c	192 ± 10 c
	K120 (K_2_SO_4_)	31.8 ± 0.8 a	29.1 ± 0.7 a	15.4 ± 0.2 a	17.2 ± 0.4 a	200 ± 13 b	211 ± 12 b
Interactions							
21 days	K_0_	35.6 ± 0.9 a	32.4 ± 0.6 a	15.1 ± 0.3 a	17.4 ± 0.2 a	181 ± 11 a	195 ± 12 a
	K286 (feldspar)	35.2 ± 0.9 a	31.8 ± 0.7 a	15.2 ± 0.3 a	17.1 ± 0.1 a	211 ± 10 a	225 ± 10 a
	K572 (feldspar)	35.4 ± 1.0 a	32.0 ± 0.8 a	15.0 ± 0.2 a	16.9 ± 0.2 a	220 ± 14 a	240 ± 11 a
	K60 (K_2_SO_4_)	35.1 ± 0.9 a	31.5 ± 0.8 a	15.4 ± 0.2 a	17.3 ± 0.2 a	203 ± 12 a	216 ± 12 a
	K120 (K_2_SO_4_)	35.6 ± 0.8 a	32.2 ± 0.6 a	15.3 ± 0.1 a	17.0 ± 0.4 a	211 ± 16 a	226 ± 12 a
28 days	K_0_	31.6 ± 0.9 a	29.4 ± 0.7 a	15.2 ± 0.2 a	17.2 ± 0.3 a	178 ± 13 a	180 ± 14 a
	K286 (feldspar)	30.9 ± 1.1 a	29.0 ± 0.7 a	15.4 ± 0.2 a	17.7 ± 0.2 a	195 ± 12 a	203 ± 13 a
	K572 (feldspar)	31.2 ± 1.0 a	28.6 ± 0.8 a	15.0 ± 0.2 a	16.9 ± 0.1 a	211 ± 16 a	229 ± 12 a
	K60 (K_2_SO_4_)	32.4 ± 0.7 a	28.1 ± 0.8 a	15.6 ± 0.3 a	17.3 ± 0.3 a	187 ± 14 a	285 ± 12 a
	K120 (K_2_SO_4_)	31.7 ± 0.8 a	29.1 ± 0.9 a	15.5 ± 0.5 a	17.5 ± 0.2 a	199 ± 10 a	207 ± 14 a
35 days	K_0_	29.2 ± 0.9 a	26.2 ± 0.8 a	15.3 ± 0.2 a	17.1 ± 0.2 a	170 ± 13 a	168 ± 12 a
	K286 (feldspar)	29.6 ± 1.0 a	26.5 ± 0.7 a	15.4 ± 0.1 a	17.5 ± 0.2 a	190 ± 12 a	197 ± 13 a
	K572 (feldspar)	28.9 ± 1.2 a	25.4 ± 0.6 a	15.1 ± 0.4 a	17.3 ± 0.3 a	202 ± 14 a	219 ± 10 a
	K60 (K_2_SO_4_)	29.7 ± 1.1 a	26.0 ± 0.8 a	15.7 ± 0.2 a	16.8 ± 0.1 a	181 ± 15 a	176 ± 11 a
	K120 (K_2_SO_4_)	28.0 ± 1.0 a	25.9 ± 1.0 a	15.3 ± 0.3 a	17.0 ± 0.2 a	189 ± 12 a	200 ± 12 a

K_0_: 0.0 kg K_2_O ha^−1^. Means followed by different letters are significant at 0.05 level of LSD. Data are means ± SD. n = 3.

**Table 3:** Effects of irrigation intervals (21, 28, and 35 days) and different potassium levels and sources (potassium sulfate or feldspar; kg ha^−1^) on vegetative parameters of fennel (*Foeniculum vulgare* Mill.) plants.

Treatments		Plant Height (cm)		Number of Branches		Stem Diameter (cm)	
		2022	2023	2022	2023	2022	2023
Irrigation intervals	21 days	167.7 ± 10 a	171.6 ± 15 a	8.11 ± 0.12 a	8.29 ± 0.09 a	1.92 ± 0.06 a	1.95 ± 0.05 a
	28 days	162.4 ± 11 b	167.2 ± 14 b	8.02 ± 0.09 b	8.13 ± 0.11 b	1.89 ± 0.08 a	1.90 ± 0.07 b
	35 days	155.5 ± 12 c	158.6 ± 12 c	6.91 ± 0.11 c	7.01 ± 0.12 c	1.75 ± 0.07 b	1.78 ± 0.06 c
K levels and sources	K_0_	152.4 ± 13 c	156.0 ± 11 c	7.01 ± 0.11 e	7.15 ± 0.11 e	1.60 ± 0.06 e	1.65 ± 0.05 d
	K286 (feldspar)	159.3 ± 14 b	162.2 ± 13 b	7.48 ± 0.10 d	7.57 ± 0.10 d	1.82 ± 0.05 d	1.86 ± 0.06 c
	K572 (feldspar)	167.9 ± 12 ab	171.6 ± 15 a	8.02 ± 0.11 b	8.14 ± 0.13 b	1.95 ± 0.06 b	1.96 ± 0.04 ab
	K60 (K_2_SO_4_)	163.6 ± 11 b	166.2 ± 14 b	7.75 ± 0.12 c	7.90 ± 0.10 c	1.87 ± 0.07 c	1.92 ± 0.06 b
	K120 (K_2_SO_4_)	170.3 ± 10 a	173.0 ± 13 a	8.14 ± 0.10 a	8.30 ± 0.11 a	2.02 ± 0.08 a	2.00 ± 0.05 a
Interactions							
21 days	K_0_	160.8 ± 12 bc	164.1 ± 12 c	7.33 ± 0.12 e	7.56 ± 0.13 d	1.63 ± 0.08 d	1.680.08 d
	K286 (feldspar)	166.9 ± 12 b	170.2 ± 10 ab	7.89 ± 0.10 d	8.00 ± 0.12 c	1.95 ± 0.08 b	1.99 ± 0.06 a
	K572 (feldspar)	170.9 ± 11 a	175.0 ± 12 a	8.44 ± 0.11 b	8.55 ± 0.14 b	1.96 ± 0.07 b	2.01 ± 0.08 a
	K60 (K_2_SO_4_)	167.0 ± 10 b	171.6 ± 12 a	8.33 ± 0.12 b	8.56 ± 0.10 b	1.97 ± 0.06 b	2.01 ± 0.08 a
	K120 (K_2_SO_4_)	173.1 ± 12 a	177.2 ± 12 a	8.56 ± 0.10 a	8.78 ± 0.11 a	2.08 ± 0.08 a	2.06 ± 0.08 a
28 days	K_0_	151.6 ± 12 cd	156.8 ± 13 d	7.22 ± 0.14 e	7.33 ± 0.11 d	1.62 ± 0.08 d	1.68 ± 0.09 d
	K286 (feldspar)	157.0 ± 10 c	162.2 ± 12 c	7.78 ± 0.11 d	7.82 ± 0.12 d	1.80 ± 0.06 c	1.85 ± 0.08 c
	K572 (feldspar)	167.2 ± 11 b	173.4 ± 14 ab	8.41 ± 0.12 b	8.54 ± 0.12 b	2.00 ± 0.07 b	1.96 ± 0.10 a
	K60 (K_2_SO_4_)	166.6 ± 12 b	170.0 ± 10 ab	8.11 ± 0.13 c	8.22 ± 0.11 c	1.91 ± 0.08 b	1.98 ± 0.06 a
	K120 (K_2_SO_4_)	169.7 ± 10 a	173.9 ± 11 ab	8.56 ± 0.12 a	8.75 ± 0.10 a	2.11 ± 0.06 a	2.03 ± 0.07 a
35 days	K_0_	144.7 ± 14 d	145.2 ± 11 e	6.44 ± 0.12 g	6.55 ± 0.12 f	1.56 ± 0.10 d	1.59 ± 0.07 e
	K286 (feldspar)	151.0 ± 11 cd	154.3 ± 12 d	6.78 ± 0.12 f	6.89 ± 0.10 e	1.72 ± 0.07 c	1.75 ± 0.08 c
	K572 (feldspar)	162.6 ± 12 b	166.5 ± 12 b	7.22 ± 0.11 e	7.33 ± 0.12 d	1.88 ± 0.08 c	1.90 ± 0.08 b
	K60 (K_2_SO_4_)	154.2 ± 13 c	157.2 ± 11 d	6.80 ± 0.10 f	6.91 ± 0.12 e	1.74 ± 0.09 c	1.77 ± 0.07 c
	K120 (K_2_SO_4_)	165.1 ± 12 b	168.0 ± 10 b	7.30 ± 0.12 e	7.36 ± 0.12 d	1.87 ± 0.08 c	1.91 ± 0.06 b

K_0_: 0.0 kg K_2_O ha^−1^. Means followed by different letters are significant at 0.05 level of LSD. Data are means ± SD. n = 3.

**Table 4:** Effects of irrigation intervals (21, 28, and 35 days) and different potassium levels and sources (potassium sulfate or feldspar; kg ha^−1^) on yield components of fennel (*Foeniculum vulgare* Mill.) plants.

Treatments		No of Umbels/Plant		No of Umbellate/Umbel		1000-Seed Weight	
		2022	2023	2022	2023	2022	2023
Irrigation intervals	21 days	40.27 ± a	42.48 ± a	60.84 ± a	64.59 ± a	12.41 ± a	12.20 ± a
	28 days	38.90 ± b	40.16 ± b	51.17 ± c	54.40 ± c	10.12 ± b	10.11 ± b
	35 days	38.94 ± b	40.67 ± b	55.55 ± b	58.47 ± b	10.98 ± b	10.79 ± b
K levels and sources	K_0_	25.56 ± d	30.19 ± c	37.79 ± c	40.43 ± c	6.62 ± c	6.25 ± c
	K286 (feldspar)	37.55 ± c	39.58 ± b	52.89 ± b	55.97 ± b	9.25 ± b	9.21 ± b
	K572 (feldspar)	42.27 ± a	43.65 ± a	64.08 ± a	66.16 ± a	12.89 ± a	12.36 ± a
	K60 (K_2_SO_4_)	39.15 ± b	40.13 ± b	54.02 ± b	56.90 ± b	9.46 ± b	9.31 ± b
	K120 (K_2_SO_4_)	43.44 ± a	44.70 ± a	65.00 ± a	67.76 ± a	13.12 ± a	12.57 ± a
Interactions							
21 days	K_0_	30.0 ± 0.4 d	35.3 ± 0.4 c	45.2 ± 0.6 d	48.1 ± 0.7 d	8.7 ± 0.1 d	8.1 ± 0.1 e
	K286 (feldspar)	40.3 ± 0.4 b	42.7 ± 0.5 a	61.3 ± 0.7 b	66.0 ± 0.8 b	12.1 ± 0.1 b	12.0 ± 0.2 b
	K572 (feldspar)	44.0 ± 0.5 a	45.0 ± 0.5 a	67.4 ± 0.7 a	70.2 ± 0.7 a	14.3 ± 0.2 a	14.1 ± 0.1 a
	K60 (K_2_SO_4_)	41.3 ± 0.5 b	43.3 ± 0.5 a	62.3 ± 0.7 b	66.4 ± 0.6 b	12.3 ± 0.1 b	12.2 ± 0.2 b
	K120 (K_2_SO_4_)	45.7 ± 0.4 a	46.1 ± 0.4 a	68.0 ± 0.6 a	72.2 ± 0.7 a	14.7 ± 0.2 a	14.6 ± 0.1 a
28 days	K_0_	25.1 ± 0.4 e	30.7 ± 0.5 d	37.0 ± 0.7 e	39.9 ± 0.6 e	6.2 ± 0.1 e	6.0 ± 0.2 f
	K286 (feldspar)	38.9 ± 0.5 c	40.2 ± 0.6 b	51.2 ± 0.8 bc	54.4 ± 0.6 bc	10.1 ± 0.2 c	10.1 ± 0.3 c
	K572 (feldspar)	43.9 ± 0.5 a	45.0 ± 0.5 a	68.4 ± 0.7 a	70.1 ± 0.7 a	14.1 ± 0.2 a	13.9 ± 0.2 a
	K60 (K_2_SO_4_)	41.3 ± 0.5 ab	41.7 ± 0.4 ab	52.0 ± 0.6 c	56.0 ± 0.7 c	10.4 ± 0.2 c	10.1 ± 0.1 c
	K120 (K_2_SO_4_)	45.5 ± 0.4 a	45.9 ± 0.5 a	69.2 ± 0.7 a	72.0 ± 0.6 a	14.2 ± 0.1 a	13.8 ± 0.2 a
35 days	K_0_	21.6 ± 0.5 e	24.6 ± 0.4 e	31.1 ± 0.6 f	33.3 ± 0.6 f	4.7 ± 0.2 f	4.7 ± 0.1 g
	K286 (feldspar)	33.4 ± 0.6 d	35.9 ± 0.4 c	46.2 ± 0.6 d	47.5 ± 0.7 d	5.5 ± 0.3 f	5.5 ± 0.2 f
	K572 (feldspar)	38.9 ± 0.5 c	41.0 ± 0.5 ab	56.5 ± 0.7 c	58.2 ± 0.7 c	10.3 ± 0.2 c	9.2 ± 0.2 d
	K60 (K_2_SO_4_)	34.9 ± 0.4 d	36.0 ± 0.5 c	47.7 ± 0.7 d	48.3 ± 0.7 d	5.7 ± 0.1 ef	5.6 ± 0.2 f
	K120 (K_2_SO_4_)	39.1 ± 0.5 c	42.1 ± 0.4 a	57.8 ± 0.6 b	59.0 ± 0.6 c	10.5 ± 0.2 c	9.3 ± 0.1 d

K_0_: 0.0 kg K_2_O ha^−1^. Means followed by different letters are significant at 0.05 level of LSD. Data are means ± SD. n = 3.

**Table 5:** Effects of irrigation intervals (21, 28, and 35 days) and different potassium levels and sources (potassium sulfate or feldspar; kg ha^−1^) on yield of fennel (*Foeniculum vulgare* Mill.) plants.

Treatments		Herb Dry Yield (kg ha^−1^)		Seed Yield (kg ha^−1^)		Biological Yield (kg ha^−1^)	
		2022	2023	2022	2023	2022	2023
Irrigation intervals	21 days	14.7 ± 0.08 a	14.9 ± 0.12 a	4.2 ± 0.01 a	4.2 ± 0.01 a	18.2 ± 0.18 a	19.1 ± 0.18 a
	28 days	13.6 ± 0.06 b	13.8 ± 0.10 b	4.0 ± 0.02 a	4.1 ± 0.01 a	17.7 ± 0.18 b	18.1 ± 0.22 b
	35 days	11.7 ± 0.07 c	11.9 ± 0.11 c	3.5 ± 0.01 b	3.7 ± 0.01 b	15.2 ± 0.19 c	15.6 ± 0.21 c
K levels and sources	K_0_	11.2 ± 0.09 c	11.5 ± 0.10 c	2.5 ± 0.01 c	2.7 ± 0.00 c	13.7 ± 0.21 d	14.1 ± 0.23 d
	K286 (feldspar)	13.2 ± 0.11 b	13.4 ± 0.09 b	3.9 ± 0.00 b	4.1 ± 0.01 b	17.1 ± 0.22 b	17.5 ± 0.19 c
	K572 (feldspar)	14.5 ± 0.10 a	14.7 ± 0.10 a	4.7 ± 0.01 a	4.5 ± 0.02 a	19.2 ± 0.18 a	19.2 ± 0.20 b
	K60 (K_2_SO_4_)	13.3 ± 0.08 b	13.4 ± 0.12 b	3.9 ± 0.02 b	4.1 ± 0.01 b	15.9 ± 0.19 c	17.6 ± 0.21 c
	K120 (K_2_SO_4_)	14.5 ± 0.09 a	14.7 ± 0.11 a	4.7 ± 0.01 a	4.5 ± 0.01 a	19.2 ± 0.21 a	19.6 ± 0.21 a
Interactions							
21 days	K_0_	12.7 ± 0.10 b	13.0 ± 0.11 b	2.8 ± 0.01 d	2.9 ± 0.01 c	15.5 ± 0.20 d	15.9 ± 0.21 c
	K286 (feldspar)	14.9 ± 0.09 a	15.0 ± 0.12 a	4.2 ± 0.01 b	4.4 ± 0.01 a	19.2 ± 0.19 b	19.4 ± 0.22 a
	K572 (feldspar)	15.3 ± 0.08 a	15.6 ± 0.10 a	5.0 ± 0.01 a	4.6 ± 0.01 a	20.3 ± 0.18 a	20.2 ± 0.20 a
	K60 (K_2_SO_4_)	15.0 ± 0.17 a	15.1 ± 0.10 a	4.3 ± 0.02 b	4.5 ± 0.01 a	15.4 ± 0.27 d	19.5 ± 0.20 a
	K120 (K_2_SO_4_)	15.4 ± 0.10 a	15.6 ± 0.19 a	5.0 ± 0.01 a	4.7 ± 0.02 a	20.4 ± 0.20 a	20.7 ± 0.29 a
28 days	K_0_	12.1 ± 0.11 c	12.4 ± 0.13 c	2.4 ± 0.01 d	2.8 ± 0.01 c	14.6 ± 0.21 d	15.2 ± 0.23 c
	K286 (feldspar)	12.7 ± 0.12 b	12.9 ± 0.09 b	4.0 ± 0.01 b	4.3 ± 0.01 a	16.9 ± 0.22 c	17.2 ± 0.19 b
	K572 (feldspar)	15.2 ± 0.10 a	15.3 ± 0.08 a	4.7 ± 0.01 a	4.5 ± 0.01 a	19.9 ± 0.20 a	19.9 ± 0.18 a
	K60 (K_2_SO_4_)	12.8 ± 0.10 b	13.0 ± 0.10 b	4.1 ± 0.01 b	4.3 ± 0.01 a	16.9 ± 0.20 c	17.5 ± 0.20 b
	K120 (K_2_SO_4_)	15.2 ± 0.09 a	15.4 ± 0.11 e	4.7 ± 0.01 a	4.6 ± 0.01 a	20.0 ± 0.19 a	20.6 ± 0.21 a
35 days	K_0_	8.8 ± 0.13 d	9.0 ± 0.11 d	2.2 ± 0.01 d	2.4 ± 0.01 c	11.0 ± 0.23 e	11.4 ± 0.21 d
	K286 (feldspar)	12.0 ± 0.09 c	12.2 ± 0.12 c	3.3 ± 0.01 c	3.6 ± 0.01 b	15.3 ± 0.19 d	15.8 ± 0.22 c
	K572 (feldspar)	12.9 ± 0.08 b	13.1 ± 0.14 b	4.3 ± 0.01 b	4.3 ± 0.01 a	17.2 ± 0.18 c	17.4 ± 0.24 b
	K60 (K_2_SO_4_)	12.1 ± 0.16 c	12.2 ± 0.10 c	3.3 ± 0.02 c	3.6 ± 0.01 b	15.4 ± 0.26 d	15.9 ± 0.20 c
	K120 (K_2_SO_4_)	12.9 ± 0.11 b	13.1 ± 0.09 b	4.4 ± 0.01 b	4.3 ± 0.01 a	17.3 ± 0.21 c	17.4 ± 0.19 b

K_0_: 0.0 kg K_2_O ha^−1^. Means followed by different letters are significant at 0.05 level of LSD. Data are means ± SD. n = 3.

**Table 6:** Effects of irrigation intervals (21, 28, and 35 days) and different potassium levels and sources (potassium sulfate or feldspar; kg ha^−1^) on oil yield of fennel (*Foeniculum vulgare* Mill.) plants.

Treatments		Oil Percent (%)		Oil Yield (L ha^−1^)	
		2022	2023	2022	2023
Irrigation intervals	21 days	1.37 ± 0.02 c	1.34 ± 0.01 b	58.31 ± 0.37 a	57.79 ± 0.47 a
	28 days	1.40 ± 0.01 b	1.35 ± 0.01 b	56.00 ± 0.39 b	57.44 ± 0.49 a
	35 days	1.43 ± 0.01 a	1.42 ± 0.02 a	50.53 ± 0.47 c	51.96 ± 0.50 b
K levels and sources	K_0_	1.36 ± 0.02 c	1.33 ± 0.03	33.46 ± 0.51 d	35.59 ± 0.51 d
	K286 (feldspar)	1.40 ± 0.01 b	1.37 ± 0.02 b	53.78 ± 0.52 c	56.15 ± 0.58 c
	K572 (feldspar)	1.42 ± 0.01 a	1.38 ± 0.02 ab	66.33 ± 0.51 a	62.55 ± 0.55 b
	K60 (K_2_SO_4_)	1.40 ± 0.02 b	1.38 ± 0.01 ab	54.30 ± 0.49 b	56.75 ± 0.59 c
	K120 (K_2_SO_4_)	1.42 ± 0.02 a	1.39 ± 0.01 a	66.85 ± 0.44 a	67.62 ± 0.61 a
Interactions					
21 days	K_0_	1.33 ± 0.01 b	1.30 ± 0.01 b	36.79 ± 0.43 e	37.37 ± 0.63 d
	K286 (feldspar)	1.37 ± 0.01 ab	1.33 ± 0.01 b	57.55 ± 0.52 c	58.95 ± 0.74 b
	K572 (feldspar)	1.39 ± 0.01 ab	1.36 ± 0.01 ab	69.50 ± 0.50 a	63.19 ± 0.72 b
	K60 (K_2_SO_4_)	1.37 ± 0.02 ab	1.34 ± 0.01 ab	58.24 ± 0.51 c	59.69 ± 0.71 b
	K120 (K_2_SO_4_)	1.38 ± 0.01 ab	1.38 ± 0.02 ab	69.45 ± 0.41 a	69.73 ± 0.62 a
28 days	K_0_	1.36 ± 0.01 ab	1.32 ± 0.01 b	33.06 ± 0.52 e	36.49 ± 0.72 d
	K286 (feldspar)	1.39 ± 0.01 ab	1.37 ± 0.01 b	56.29 ± 0.63 c	58.45 ± 0.86 b
	K572 (feldspar)	1.41 ± 0.01 a	1.35 ± 0.01 ab	66.50 ± 0.54 a	62.69 ± 0.75 b
	K60 (K_2_SO_4_)	1.40 ± 0.01 ab	1.38 ± 0.01 ab	57.00 ± 0.42 c	59.12 ± 0.64 b
	K120 (K_2_SO_4_)	1.42 ± 0.01 a	1.35 ± 0.01 ab	67.14 ± 0.53 a	70.47 ± 0.73 a
35 days	K_0_	1.40 ± 0.01 ab	1.38 ± 0.01 ab	30.54 ± 0.43 e	32.92 ± 0.61 d
	K286 (feldspar)	1.43 ± 0.01 a	1.41 ± 0.01 a	47.50 ± 0.45 d	51.05 ± 0.62 c
	K572 (feldspar)	1.45 ± 0.01 a	1.44 ± 0.01 a	63.00 ± 0.24 b	61.76 ± 0.72 b
	K60 (K_2_SO_4_)	1.43 ± 0.02 a	1.41 ± 0.01 a	47.65 ± 0.53 d	51.43 ± 0.73 c
	K120 (K_2_SO_4_)	1.46 ± 0.01 a	1.45 ± 0.01 a	63.97 ± 0.42 b	62.67 ± 0.62 b

K_0_: 0.0 kg K_2_O ha^−1^. Means followed by different letters are significant at 0.05 level of LSD. Data are means ± SD. n = 3.

**Table 7:** Effects of irrigation intervals (21, 28, and 35 days) and different potassium levels and sources (potassium sulfate or feldspar; kg ha^−1^) on NPK contents in seeds of fennel (*Foeniculum vulgare* Mill.) plants.

Treatments		N-Seed (%)		P-Seed (%)		K-Seed (%)	
		2022	2023	2022	2023	2022	2023
Irrigation intervals	21 days	2.75 ± 0.02 a	2.62 ± 0.03 a	0.33 ± 0.01 a	0.31 ± 0.02 a	2.40 ± 0.02 a	2.37 ± 0.02 a
	28 days	2.60 ± 0.01 b	2.45 ± 0.02 b	0.28 ± 0.01 b	0.27 ± 0.01 b	2.31 ± 0.04 b	2.30 ± 0.05 b
	35 days	2.43 ± 0.02 c	2.27 ± 0.03 c	0.23 ± 0.01 c	0.21 ± 0.01 c	2.25 ± 0.03 c	2.23 ± 0.04 c
K levels and sources	K_0_	2.29 ± 0.01 c	2.20 ± 0.02 c	0.22 ± 0.01 c	0.20 ± 0.01 c	2.24 ± 0.02 c	2.22 ± 0.03 c
	K286 (feldspar)	2.54 ± 0.02 b	2.38 ± 0.02 b	0.26 ± 0.02 b	0.25 ± 0.01 b	2.30 ± 0.02 b	2.28 ± 0.03 b
	K572 (feldspar)	2.76 ± 0.04 a	2.61 ± 0.03 a	0.31 ± 0.01 a	0.30 ± 0.02 a	2.35 ± 0.02 ab	2.35 ± 0.04 a
	K60 (K_2_SO_4_)	2.57 ± 0.03 b	2.41 ± 0.04 b	0.28 ± 0.00 b	0.27 ± 0.01 b	2.31 ± 0.03 b	2.29 ± 0.03 b
	K120 (K_2_SO_4_)	2.81 ± 0.02 a	2.64 ± 0.03 a	0.33 ± 0.01 a	0.31 ± 0.01 a	2.38 ± 0.04 a	2.36 ± 0.02 a
Interactions							
21 days	K_0_	2.55 ± 0.04 d	2.41 ± 0.06 c	0.26 ± 0.01 b	0.24 ± 0.01 b	2.31 ± 0.05 b	2.29 ± 0.07 c
	K286 (feldspar)	2.67 ± 0.04 c	2.56 ± 0.06 b	0.31 ± 0.01 a	0.30 ± 0.01 ab	2.39 ± 0.06 a	2.36 ± 0.08 b
	K572 (feldspar)	2.89 ± 0.05 a	2.77 ± 0.07 a	0.37 ± 0.01 a	0.35 ± 0.01 a	2.44 ± 0.05 a	2.42 ± 0.07 a
	K60 (K_2_SO_4_)	2.70 ± 0.05 b	2.59 ± 0.07 b	0.33 ± 0.02 a	0.31 ± 0.01 a	2.40 ± 0.04 a	2.37 ± 0.06 b
	K120 (K_2_SO_4_)	2.93 ± 0.04 a	2.78 ± 0.06 a	0.39 ± 0.01 a	0.37 ± 0.02 a	2.46 ± 0.05 a	2.43 ± 0.07 a
28 days	K_0_	2.31 ± 0.05 d	2.27 ± 0.07 d	0.22 ± 0.01 c	0.20 ± 0.01 b	2.26 ± 0.04 c	2.24 ± 0.06 c
	K286 (feldspar)	2.59 ± 0.06 c	2.40 ± 0.08 c	0.27 ± 0.01 b	0.26 ± 0.01 b	2.29 ± 0.05 b	2.27 ± 0.07 c
	K572 (feldspar)	2.72 ± 0.05 b	2.56 ± 0.07 b	0.31 ± 0.01 a	0.30 ± 0.01 ab	2.35 ± 0.05 b	2.34 ± 0.07 b
	K60 (K_2_SO_4_)	2.62 ± 0.04 c	2.44 ± 0.06 c	0.29 ± 0.01 b	0.27 ± 0.01 b	2.29 ± 0.05 b	2.28 ± 0.07 c
	K120 (K_2_SO_4_)	2.78 ± 0.05 b	2.59 ± 0.07 b	0.33 ± 0.01 a	0.31 ± 0.01 ab	2.36 ± 0.04 b	2.35 ± 0.06 b
35 days	K_0_	2.01 ± 0.04 e	1.92 ± 0.06 e	0.18 ± 0.01 c	0.16 ± 0.01 c	2.15 ± 0.04 d	2.13 ± 0.06 d
	K286 (feldspar)	2.35 ± 0.05 d	2.18 ± 0.07 d	0.21 ± 0.01 c	0.20 ± 0.01 b	2.23 ± 0.04 c	2.21 ± 0.06 c
	K572 (feldspar)	2.68 ± 0.05 b	2.50 ± 0.07 b	0.25 ± 0.01 b	0.24 ± 0.01 b	2.30 ± 0.05 b	2.29 ± 0.07 c
	K60 (K_2_SO_4_)	2.39 ± 0.05 d	2.21 ± 0.07 d	0.22 ± 0.02 c	0.22 ± 0.01 b	2.25 ± 0.05 c	2.23 ± 0.07 c
	K120 (K_2_SO_4_)	2.71 ± 0.04 b	2.55 ± 0.06 b	0.27 ± 0.01 b	0.25 ± 0.01 b	2.31 ± 0.04 b	2.30 ± 0.06 b

K_0_: 0.0 kg K_2_O ha^−1^. Means followed by different letters are significant at 0.05 level of LSD. Data are means ± SD. n = 3.

**Table 8:** Effects of irrigation intervals (21, 28, and 35 days) and different potassium levels and sources (potassium sulfate or feldspar; kg ha^−1^) on NPK contents in herb of fennel (*Foeniculum vulgare* Mill.) plants.

Treatments		N-Herb (%)		P-Herb (%)		K-Herb (%)	
		2022	2023	2022	2023	2022	2023
Irrigation intervals	21 days	1.54 ± 0.03 a	1.52 ± 0.03 a	0.33 ± 0.01 a	0.31 ± 0.01 a	2.22 ± 0.03 a	2.20 ± 0.03 a
	28 days	1.46 ± 0.02 b	1.44 ± 0.02 b	0.31 ± 0.01 b	0.28 ± 0.01 b	2.19 ± 0.03 b	2.17 ± 0.03 b
	35 days	1.42 ± 0.04 c	1.39 ± 0.03 c	0.28 ± 0.02 c	0.27 ± 0.01 c	2.16 ± 0.04 c	2.14 ± 0.02 c
K levels and sources	K_0_	1.40 ± 0.01 d	1.37 ± 0.02 d	0.24 ± 0.01 e	0.23 ± 0.02 c	2.13 ± 0.03 c	2.11 ± 0.04 c
	K286 (feldspar)	1.44 ± 0.03 c	1.42 ± 0.03 c	0.30 ± 0.01 d	0.28 ± 0.01 b	2.18 ± 0.02 b	2.16 ± 0.03 b
	K572 (feldspar)	1.52 ± 0.02 b	1.49 ± 0.04 b	0.33 ± 0.02 b	0.31 ± 0.02 a	2.22 ± 0.05 ab	2.20 ± 0.03 a
	K60 (K_2_SO_4_)	1.46 ± 0.03 c	1.43 ± 0.03 c	0.31 ± 0.00 c	0.28 ± 0.01 b	2.19 ± 0.03 b	2.17 ± 0.04 b
	K120 (K_2_SO_4_)	1.55 ± 0.04 a	1.55 ± 0.02 a	0.35 ± 0.01 a	0.32 ± 0.01 a	2.23 ± 0.04 a	2.21 ± 0.05 a
Interactions							
21 days	K_0_	1.43 ± 0.04 c	1.40 ± 0.06 c	0.27 ± 0.01 b	0.27 ± 0.01 b	2.16 ± 0.04 b	2.14 ± 0.06 b
	K286 (feldspar)	1.48 ± 0.05 c	1.46 ± 0.07 c	0.33 ± 0.01 a	0.30 ± 0.01 a	2.21 ± 0.05 a	2.19 ± 0.07 a
	K572 (feldspar)	1.62 ± 0.05 a	1.58 ± 0.07 b	0.36 ± 0.01 a	0.34 ± 0.01 a	2.25 ± 0.05 a	2.23 ± 0.07 a
	K60 (K_2_SO_4_)	1.51 ± 0.05 b	1.48 ± 0.07 c	0.34 ± 0.02 a	0.30 ± 0.01 a	2.21 ± 0.05 a	2.20 ± 0.07 a
	K120 (K_2_SO_4_)	1.64 ± 0.04 a	1.67 ± 0.06 a	0.37 ± 0.01 a	0.35 ± 0.02 a	2.26 ± 0.04 a	2.24 ± 0.06 a
28 days	K_0_	1.39 ± 0.04 d	1.36 ± 0.06 d	0.24 ± 0.01 c	0.23 ± 0.01 c	2.13 ± 0.06 b	2.11 ± 0.07 b
	K286 (feldspar)	1.44 ± 0.04 c	1.41 ± 0.06 c	0.29 ± 0.01 b	0.27 ± 0.01 b	2.18 ± 0.06 ab	2.16 ± 0.08 a
	K572 (feldspar)	1.50 ± 0.05 bc	1.46 ± 0.07 c	0.34 ± 0.01 a	0.30 ± 0.01 a	2.22 ± 0.05 a	2.20 ± 0.07 a
	K60 (K_2_SO_4_)	1.46 ± 0.05 c	1.42 ± 0.07 c	0.31 ± 0.01 a	0.28 ± 0.01 b	2.19 ± 0.04 a	2.17 ± 0.06 a
	K120 (K_2_SO_4_)	1.53 ± 0.04 b	1.54 ± 0.06 b	0.35 ± 0.01 a	0.31 ± 0.01 a	2.23 ± 0.05 a	2.20 ± 0.07 a
35 days	K_0_	1.37 ± 0.05 d	1.34 ± 0.07 d	0.21 ± 0.01 c	0.20 ± 0.01 c	2.10 ± 0.04 b	2.08 ± 0.06 b
	K286 (feldspar)	1.39 ± 0.06 d	1.38 ± 0.08 d	0.27 ± 0.01 b	0.26 ± 0.01 b	2.16 ± 0.04 b	2.13 ± 0.06 b
	K572 (feldspar)	1.45 ± 0.05 c	1.42 ± 0.07 c	0.30 ± 0.01 a	0.29 ± 0.01 a	2.19 ± 0.05 a	2.18 ± 0.07 a
	K60 (K_2_SO_4_)	1.41 ± 0.04 c	1.39 ± 0.06 d	0.28 ± 0.02 b	0.27 ± 0.01 b	2.17 ± 0.05 b	2.13 ± 0.07 b
	K120 (K_2_SO_4_)	1.47 ± 0.05 c	1.44 ± 0.07 c	0.32 ± 0.01 a	0.31 ± 0.01 a	2.20 ± 0.04 a	2.19 ± 0.06 a

K_0_: 0.0 kg K_2_O ha^−1^. Means followed by different letters are significant at 0.05 level of LSD. Data are means ± SD. n = 3.

**Table 9:** Meteorological data during the experiment in 2022 and 2023.

Month	Minimum Temperature (°C)	Maximum Temperature (°C)	Precipitation (mm)
December 2021	20.5	8.5	0
January 2022	19.5	7.5	1
February 2022	21.5	8.5	0.5
December 2022	20.9	7.4	0
January 2023	17.5	8.5	1
February 203	22.0	8.5	0

## Data Availability

Data will be made available upon the request.

## References

[1] Mohamed M., Ali A., Ibrahim M. (2022). Fennel (*Foeniculum vulgare* Mill.) Growth, Productivity and Essential Oil Yield under Different Sowing Methods and Some Stimulant Substances. Arch. Agric. Sci. J..

[2] Jadid N., Widodo A.F., Ermavitalini D., Sa’adah N.N., Gunawan S., Nisa C. (2023). The Medicinal Umbelliferae Plant Fennel (*Foeniculum vulgare* Mill.): Cultivation, Traditional Uses, Phytopharmacological Properties, and Application in Animal Husbandry. Arab. J. Chem..

[3] Rani S.N., Aslam Z.N., Babar S.N., Musaddiq S., Fayyaz I., Mustafa K. (2024). *Foeniculum vulgare* (Fennel): A Versatile Herb With Culinary, Medicinal, and Environmental Significance. Therapeutic and Pharmacological Applications of Ethnobotany.

[4] Radwan E.M.A., Hassan E.A., Abdelal O.A., El-Gohary A.I. (2024). Enhancing the Productivity of Fennel (*Foeniculum vulgare* Miller) Plants by Using Some Organic and Bio Fertilizers Treatments. New Val. J. Agric. Sci..

[5] Radwan E.M.A., Hassan E.A., Abdelal O.A., El-Gohary A.I., El-Ramady H., Alshaal T., Bakr N., Elbana T., Mohamed E., Belal A.-A. (2019). The Soils of Egypt.

[6] Azizi M.A., Leandro J. (2025). Factors Affecting Transboundary Water Disputes: Nile, Indus, and Euphrates–Tigris River Basins. Water.

[7] Fereres E., Soriano M.A. (2006). Deficit Irrigation for Reducing Agricultural Water Use. J. Exp. Bot..

[8] Telci I., Demirtas I., Sahin A. (2009). Variation in Plant Properties and Essential Oil Composition of Sweet Fennel (*Foeniculum vulgare* Mill.) Fruits during Stages of Maturity. Ind. Crops Prod..

[9] Geerts S., Raes D. (2009). Deficit Irrigation as an On-Farm Strategy to Maximize Crop Water Productivity in Dry Areas. Agric. Water Manag..

[10] Mahiwal S., Pandey G.K. (2022). Potassium: A Vital Nutrient Mediating Stress Tolerance in Plants. J. Plant Biochem. Biotechnol..

[11] Mohammed K., Eid M., Usman A. (2024). Enhancing the Agronomic Performance of Potassium Fertilizer and Potassium-Bearing Minerals in Sandy Loam Soil by Adding Humic Acids and Mycorrhiza. Assiut J. Agric. Sci..

[12] Richards L.A. (1954). Diagnosis and Improvement of Saline Alkali Soils, Agriculture.

[13] Marschner P., Rengel Z. (2023). Nutrient Availability in Soils. Marschner’s Mineral Nutrition of Plants.

[14] Tinker P.B., Nye P. (2000). Solute Movement in the Rhizosphere.

[15] Jungk A.O. (2002). Dynamics of Nutrient Movement at the Soil-Root Interface. Plant Roots: The Hidden Half.

[16] Kumar P., Kumar T., Singh S., Tuteja N., Prasad R., Singh J. (2020). Potassium: A Key Modulator for Cell Homeostasis. J. Biotechnol..

[17] Abou-el-Seoud I.I., Abdel-Megeed A. (2012). Impact of Rock Materials and Biofertilizations on P and K Availability for Maize (*Zea maize*) under Calcareous Soil Conditions. Saudi J. Biol. Sci..

[18] Hu W., Lu Z., Meng F., Li X., Cong R., Ren T., Sharkey T.D., Lu J. (2020). The Reduction in Leaf Area Precedes That in Photosynthesis under Potassium Deficiency: The Importance of Leaf Anatomy. New Phytol..

[19] Parmoon G., Ebadi A., Hashemi M., Hawrylak-Nowak B., Baskin C., Jahanbakhsh S. (2022). Plant Growth Regulators Improve Grain Production and Water Use Efficiency of *Foeniculum vulgare* Mill. under Water Stress. Plants.

[20] Emami Bistgani Z., Siadat S.A., Bakhshandeh A., Ghasemi Pirbalouti A., Hashemi M. (2017). Interactive Effects of Drought Stress and Chitosan Application on Physiological Characteristics and Essential Oil Yield of *Thymus daenensis* Celak. Crop J..

[21] Johnson R., Vishwakarma K., Hossen S., Kumar V., Shackira A.M., Puthur J.T., Abdi G., Sarraf M., Hasanuzzaman M. (2022). Potassium in Plants: Growth Regulation, Signaling, and Environmental Stress Tolerance. Plant Physiol. Biochem..

[22] Mekawy A.Y., El-Hafeez A.M.A. (2020). Reducing the Amount of Mineral Phosphorus and Potassium Fertilizers by Using Its Natural Sources for Red Globe Grapevines. J. Appl. Hortic..

[23] Bhardwaj S., Kapoor B., Kapoor D., Thakur U., Dolma Y., Raza A. (2025). Manifold Roles of Potassium in Mediating Drought Tolerance in Plants and Its Underlying Mechanisms. Plant Sci..

[24] Cochrane T.T., Cochrane T.A. (2009). The Vital Role of Potassium in the Osmotic Mechanism of Stomata Aperture Modulation and Its Link with Potassium Deficiency. Plant Signal. Behav..

[25] Mostofa M.G., Rahman M., Ghosh T.K., Kabir A.H., Abdelrahman M., Rahman Khan A., Mochida K., Tran L.-S.P. (2022). Potassium in Plant Physiological Adaptation to Abiotic Stresses. Plant Physiol. Biochem..

[26] Hafiz Y., Ewis M. (2015). Effect of Irrigation Regime and Potassium Fertilizer Rates on Growth, Yield, Oil Composition and Some Water Relations of Fennel Plant (*Foeniculum vulgare* Mill.) under Middle Egypt Conditions. Egypt. J. Agric. Sci..

[27] Ali H., Ahmed N., Abu-Hashim M. (2020). Potential Effect of Irrigation Intervals and Potassium Phthalate on Fennel Plants Grown in Semi-Arid Regions. Egypt. J. Soil Sci..

[28] Latimer G.W., AOAC (2016). Official Methods of Analysis of AOAC International.

[29] WRB (2024). World Reference Base for Soil Resources. International Soil Classification System for Naming Soils and Creating Legends for Soil Maps.

[30] Sparks D.L., Page A.L., Helmke P.A., Loeppert R.H. (1996). Methods of Soil Analysis. Part 3: Chemical Methods.

[31] Olsen S.R., Cole C.V., Watanabe F.S. (1954). Estimation of Available Phosphorus in Soils by Extraction with Sodium Bicarbonate.

[32] van Schouwenburg J.C., Walinga I. (1967). The Rapid Determination of Phosphorus in Presence of Arsenic, Silicon and Germanium. Anal. Chim. Acta.

[33] Jackson M.L. (1973). Soil Chemical Analysis.

